# Synthesis of Novel Methyl 7-[(Hetero)arylamino]thieno[2,3-*b*]pyrazine-6-carboxylates and Antitumor Activity Evaluation: Effects in Human Tumor Cells Growth, Cell Cycle Analysis, Apoptosis and Toxicity in Non-Tumor Cells

**DOI:** 10.3390/molecules26164823

**Published:** 2021-08-10

**Authors:** Juliana M. Rodrigues, Ricardo C. Calhelha, António Nogueira, Isabel C. F. R. Ferreira, Lillian Barros, Maria-João R. P. Queiroz

**Affiliations:** 1 Centro de Química, Universidade do Minho (CQUM), Campus de Gualtar, 4710-057 Braga, Portugal; id7018@alunos.uminho.pt; 2 Centro de Investigação de Montanha (CIMO), Instituto Politécnico de Bragança, Campus de Santa Apolónia, 5300-253 Bragança, Portugal; calhelha@ipb.pt (R.C.C.); ajmnogueira@ipb.pt (A.N.); iferreira@ipb.pt (I.C.F.R.F.); Lillian@ipb.pt (L.B.)

**Keywords:** C–N Buchwald–Hartwig coupling, thieno[2,3-*b*]pyrazines, antitumor activity, gastric adenocarcinoma, cell cycle, apoptosis

## Abstract

Several novel methyl 7-[(hetero)arylamino]thieno[2,3-*b*]pyrazine-6-carboxylates were synthesized by Pd-catalyzed C–N Buchwald–Hartwig cross-coupling of either methyl 7-aminothieno[3,2-*b*]pyrazine-6-carboxylate with (hetero)arylhalides or 7-bromothieno[2,3-*b*]pyrazine-6-carboxylate with (hetero)arylamines in good-to-excellent yields (50% quantitative yield), using different reaction conditions, namely ligands and solvents, due to the different electronic character of the substrates. The antitumoral potential of these compounds was evaluated in four human tumor cell lines: gastric adenocarcinoma (AGS), colorectal adenocarcinoma (CaCo-2), breast carcinoma (MCF7), and non-small-cell lung carcinoma (NCI-H460) using the SRB assay, and it was possible to establish some structure–activity relationships. Furthermore, they did not show relevant toxicity against a non-tumor cell line culture from the African green monkey kidney (Vero). The most promising compounds (GI_50_ ≤ 11 µM), showed some selectivity either against AGS or CaCo-2 cell lines without toxicity at their GI_50_ values. The effects of the methoxylated compounds **2b** (2-OMeC_6_H_4_), **2f** and **2g** (3,4- or 3,5-diOMeC_6_H_3_, respectively) on the cell cycle profile and induction of apoptosis were further studied in the AGS cell line. Nevertheless, even for the most active (GI_50_ = 7.8 µM) and selective compound (**2g**) against this cell line, it was observed that a huge number of dead cells gave rise to an atypical distribution on the cell cycle profile and that these cells were not apoptotic, which points to a different mechanism of action for the AGS cell growth inhibition.

## 1. Introduction

The Pd-catalyzed amination of aryl halides has become a fundamental tool in the synthesis of di(hetero)arylamines over the past two decades [1,2,3,4,5,6,7,8,9,10]. As this type of compounds plays important roles in the development of pharmaceuticals, agrochemicals, and organic compounds for materials science, the scope of application of the C–N Buchwald–Hartwig cross-coupling was developed and improved, using different ligands, bases, and catalysts, which promoted general methodologies that find applications either in academic research or industry. The choice of the catalyst system is largely dependent on the geometric and electronic character of the substrates. The use of different bases allowed the development of stronger or milder conditions depending on the functional moieties in the substrates. Over the years, some generations of ligands were developed: the monodentate phosphines PAr_3_-type or PR_3_ that have often been employed; the bidentate phosphines including the most commonly used *rac*-BINAP and Xantphos; and the dialkylbiarylphosphines that due to their structural variability can be tuned to promote the desired reactivity or selectivity [11,12,13].

The thieno[2,3-*b*]pyrazine skeleton has been found in natural products such as urothion and its derivatives [14] and in biologically active synthetic compounds. Some derivatives have been described as selective inhibitors of serine/threonine kinase 4 associated with interleukine-1 receptor (IRAK 4) [15], as inhibitors of ubiquitin-specific protease 28 (USP 28) and/or USP 25 [16], and as serine/threonine kinase B-Raf inhibitors [17], all useful in the prevention or treatment of inflammation, cell proliferation, and immune-related conditions and disease.

Our research group had already applied the C–N Buchwald–Hartwig cross-coupling to the synthesis of di(hetero)arylthieno[3,2-*b*]pyridines either functionalizing the pyridine [18,19] or the thiophene ring [20], with some of them showing to be promising as potential antitumor compounds.

In this work, due to the biological relevance of the thieno[2,3-*b*]pyrazine moiety and of di(hetero)arilamines in general, a series of novel methyl 7-[(hetero)arylamino]thieno[2,3-*b*]pyrazine-6-carboxylates were synthesized by Pd-catalyzed C–N Buchwald–Hartwig cross-coupling of either methyl 7-aminothieno[3,2-*b*]pyrazine-6-carboxylate with (hetero)arylhalides or 7-bromothieno[2,3-*b*]pyrazine-6-carboxylate with (hetero)arylamines in good-to-excellent yields, using different ligands and solvents, taking into account the electronic character of the coupling components. The antitumoral potential of the di(hetero)arylamines prepared against four human tumor cell lines and their toxicity using a non-tumor cell line were evaluated. The most promising compounds were submitted to cell cycle analysis and apoptosis induction studies in one of the cell lines studied.

## 2. Results and Discussion

### 2.1. Synthesis of Methyl 7-[(Hetero)arylamino]thieno[2,3-b]pyrazine-6-carboxylates ***2a***–***2o***

**Scheme 1:**
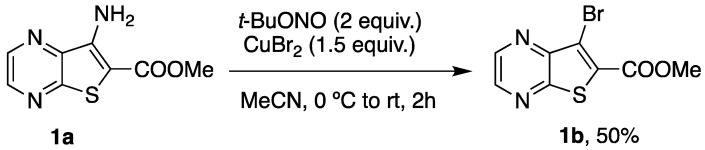
Synthesis of methyl 7-bromothieno[2,3-*b*]pyrazine-2-carboxylate (**1b**) from amine **1a**.

The coupling component methyl 7-aminothieno[2,3-*b*]pyrazine-2-carboxylate **1a** was prepared following the procedure previously described by Peinador et al. [21]. Compound **1a** was treated with *t*-butylnitrite (*t*-BuONO) and CuBr_2_ in acetonitrile at room temperature, for 2h, to obtain the methyl 7-bromothieno[2,3-*b*]pyrazine-2-carboxylate **1b** in 50% yield as another coupling component, following the reaction conditions earlier applied by us for the synthesis of a brominated thieno[3,2-*b*]pyridine [22] (Scheme 1). 

The coupling components **1a** and **1b** were reacted with (hetero)arylhalides or (hetero)arylamines, respectively. Based on our research group experience [18] and that of others [23] in C–N Buchwald–Hartwig couplings, deactivated amine **1a** (bearing an EWG in the adjacent position) as coupling component, Xantphos as ligand, Cs_2_CO_3_ as the base, and 1,4-dioxane as solvent were used (Table 1, reaction conditions A), while withthe activated bromo compound **1b**, for the cross-coupling reaction, *rac*-BINAP as the ligand, Cs_2_CO_3_ as the base, and toluene as solvent were used (Table 1, reaction conditions B). With these different conditions, taking into account the electronic character of the substrates, the corresponding di(hetero)arylamines **2a**–**2o** were thus obtained in good-to-excellent yields. 

From analysis of Table 1, it can be observed that the presence of either one methoxy group or a fluor atom in the anilines, in the coupling with **1b**, gave the corresponding di(hetero)arylamines **2b**–**2d** and **2i**–**2k**, in good-to-high yields using reaction conditions B (entries 2–4 and 9–11). The di- and trimethoxylated anilines reacting with **1b**, in the same conditions, gave the corresponding di(hetero)arylamines **2e**–**2h** in high-to-excellent yields due to the high activation of both substrates for the C–N cross-coupling (entries 5–8). The reaction of amine **1a** with the activated *p*-bromobenzonitrile gave compound **2n** in quantitative yield using reaction conditions A (entry 14). Nevertheless, the couplings of the deactivated amine **1a** using reaction conditions A, with bromobenzene, 3-bromopyridine, and 2-bromonitrobenzene gave the corresponding products **2a**, **2l**, and **2o** only in good yields (entries 1, 12, and 15). The formation of diheteroarylamine **2m** from **1b** and pyrrole in 71% yield (entry 13) was also notable.

### 2.2. Cell Growth Inhibitory Effect of Compounds ***2a***–***2o*** on AGS, CaCo-2, MCF7, NCI-H460 Cell Lines and on a Non-Tumor Cell Line (Vero) 

The antitumor potential of the di(hetero)arylamines **2a***–***2o** was evaluated using the sulforhodamine B (SRB) assay [24,25] to establish some structure–activity relationships. Four human tumor cell lines (acquired from Leibniz-Institut DSMZ) were used: gastric adenocarcinoma (AGS), colorectal adenocarcinoma (CaCo-2), breast adenocarcinoma (MCF7), and non-small-cell lung cancer (NCI-H460), as well as a non-tumor cell line from African green monkey kidney (Vero) to evaluate the toxicity of the compounds. Ellipticine was used as a positive control, and the results are presented in GI_50_ values (µM) (Table 2). 

The results attained (Table 2) allow us to identify some promising antitumor compounds (GI_50_ ≤ 11 µM), namely against AGS and CaCo-2 cell lines. Compounds **2f**, **2h**, and **2n** showed lower GI_50_ values against CaCo-2 (8, 9.2, and 10.9 µM, respectively), **2h** and **2n** being selective for this cell line among the cell lines used. The presence of a cyano group in the *para*-position of the phenyl ring (**2n**) led to a decrease in the GI_50_ value (10.9 µM) comparing with compound **2a** with a non-substituted phenyl ring (38 µM). It is noteworthy that for the CaCo-2 cell line, the lowest GI_50_ values were obtained for di- and tri-methoxylated compounds **2f** (8 µM) and **2h** (9.2 µM), bearing simultaneous methoxy groups in the 3 and 4 positions on the phenyl ring relative to the amine, which seems to be an important feature for the inhibition of cell growth in this cell line.

Regarding the AGS cell line, the presence of methoxy groups is crucial for the cell growth inhibition, as observed for compounds **2b** (2-OMeC_6_H_4_, 9.8 µM), **2f** (3,4-diOMeC_6_H_3_, 9.2 µM), and **2g** (3,5-diOMeC_6_H_3_, 7.8 µM). Compounds **2b** and **2g** were selective for this cell line, while compound **2f** presented low GI_50_ values (<10 µM) against both AGS and CaCo-2 cell lines (Table 2). 

Notably, given the GI_50_ values presented for compound **2** in the AGS and CaCo-2 cell lines, they did not show relevant toxicity in the Vero non-tumor cell line presenting higher GI_50_ values. Despite the very low GI_50_ values (≤1 µM) for the positive control Ellipticine in the human tumor cell lines tested, this is also toxic for the non-tumor line presenting a GI_50_ = 0.6 µM (Table 2).

Compound **2g** showed to be the most promising one due to its selectivity against the AGS cell line and the lowest GI_50_ value presented (7.8 µM), together with the lower toxicity for the Vero cell line (GI_50_ = 144 µM).

With these results in hand, AGS cell cycle profile effects and induction of apoptosis studies for compounds **2b**, **2f**, and **2g** were performed.

### 2.3. Effects of Compounds ***2b***, ***2f***, and ***2g*** on AGS Cell Cycle Profile

**Figure 1:**
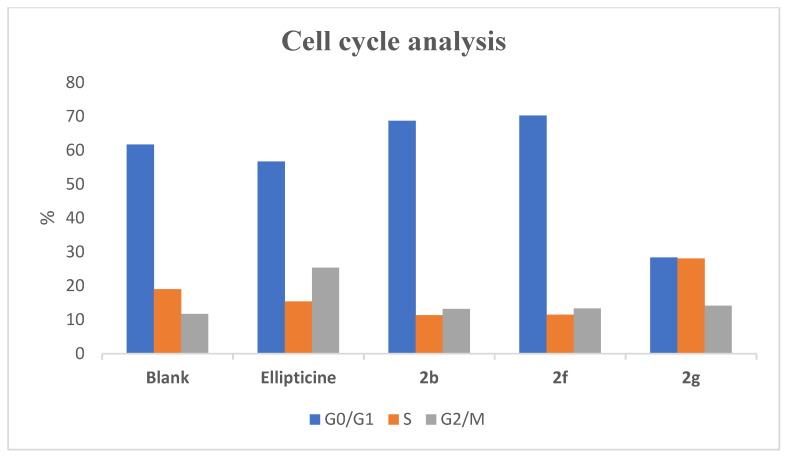
Effects of compounds **2b**, **2f**, and **2g** on AGS cell cycle profile at their GI_50_ concentrations.

AGS cell cycle analysis was carried out using propidium iodide (PI) staining and flow cytometry [26] for compounds **2b**, **2f**, and **2g** at their GI_50_ concentrations (Table 2). This assay is based on the measurement of the DNA content in the PI-labeled nuclei. The results are presented in Figure 1.

Compounds **2b** and **2f** caused cell cycle arrest in G0/G1 phases, although this result was not statistically significant (Qi2 test). On the other hand, for the G2/M phase, these compounds did not present any difference in the percentage of cells compared to the blank. 

**Figure 2:**
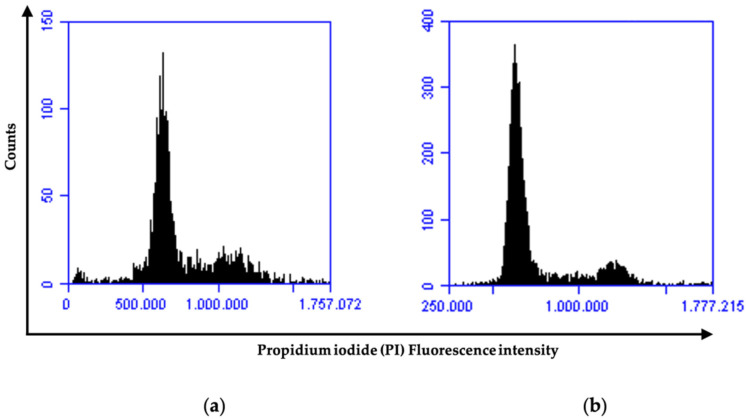
Cell cycle of blank (**a**) and compound **2f** (**b**). Note that 250.000; 500.000; 1.000.000; 1.757.072 and 1.777.215 correspond to 250,000; 500,000; 1,000,000; 1,757,072; 1,777,215.

In Figure 2, histograms of the AGS cell cycle profile for blank and compound **2f** are presented. Compound **2b** presented a similar histogram to the one obtained for the blank, and compound **2g** caused a high percentage of cell death and an atypical distribution on cell cycle profile (results not shown).

### 2.4. Effect of Compounds ***2b***, ***2f***, and ***2g*** on Induction of Apoptosis in AGS Cell Line

**Figure 3:**
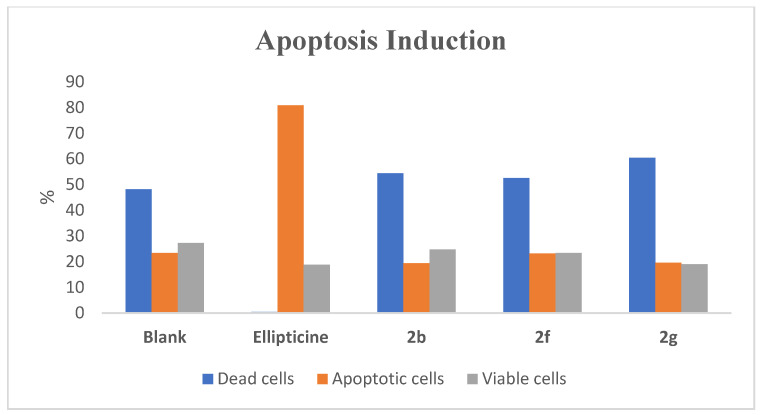
Induction of apoptotic process by compounds **2b**, **2f**, and **2g** at their GI_50_ values in AGS cell line.

Apoptosis induction was performed using the Fluorescein Isothiocyanate (FITC) Annexin V Apoptosis Kit (BD Biosciences, San Jose, CA, USA), and measured by flow cytometry for compounds **2b**, **2f**, and **2g** (Figure 3). FITC Annexin V staining was used to determine the percentage of cells within a population that are actively undergoing apoptosis [27]. 

**Figure 4:**
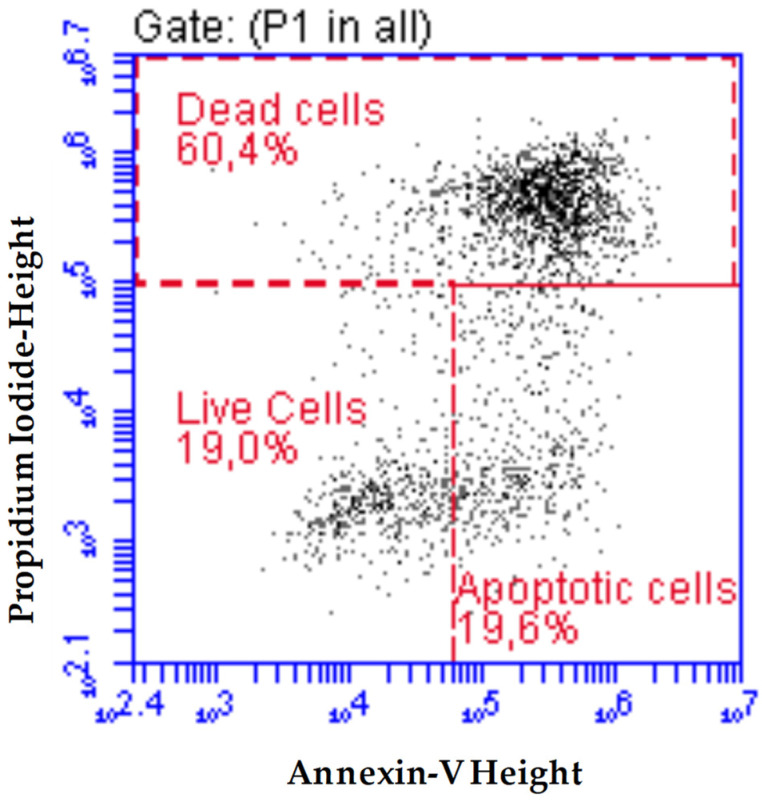
Compound **2g** apoptosis induction.

Regarding the apoptotic process, compound **2g** caused a large amount of cell death (Figure 3 and Figure 4), which was in a similar range to what was observed in the cell cycle studies. In addition, compounds **2b** and **2f** also caused a moderated cell death compared to the blank (Figure 3). 

For the tested compounds (**2b**, **2f**, and **2g**), a high number of cells in apoptosis was expected, due to the GI_50_ values obtained for the AGS cell line (Table 2). However, this was not verified, which led us to conclude that cytotoxicity against the AGS cell line involves mechanisms other than apoptosis.

## 3. Materials and Methods

### 3.1. Chemistry

Melting points (°C) were determined in a SMP3 Stuart apparatus. ^1^H, ^13^C, and ^19^F NMR spectra were recorded on a Bruker Advance III (Bruker, Bremen, Germany) at 400, 100.6, and 376.48 MHz, respectively (see Supplementary Materials), using the signals of the non-deuterated solvents of CHCl_3_ (7.27 ppm) of the CDCl_3_ or of DMSO (2.49 ppm) of the DMSO-*d_6_*, as internal reference relatively to TMS (0 ppm). DEPT (θ = 135°) and bi-dimensional homo ^1^H–^1^H (COSY) and heteronuclear correlations ^1^H-^13^C (HMQC and HMBC) were used to attribute some signals. HRMS were obtained at the external service of mass spectrometry of the University of Vigo using EI M^+^ or ESI [M + H]^+^. Reactions were followed by thin-layer chromatography (TLC). Dry flash column chromatography on silica gel 0.035–0.070 mm, 60 A and Celite^®^ 545 was used and this can be followed by column chromatography using solvent gradient to purify the compounds. Petroleum ether refers to the boiling range 40–60 °C. Ether refers to diethyl ether.

#### 3.1.1. Synthesis of Methyl 7-Bromothieno[2,3-b]pyrazine-6-carboxylate (**1b**)

*t*-Butyl nitrite (2.86 mmol, 341.3 μL) was added to a solution of CuBr_2_ (2.15 mmol, 0.480 g) in acetonitrile (5 mL) at 0 °C, followed by addition of amine **1a** (1.43 mmol, 0.300 g) in portions (three times, 5 min between each addition). The mixture was stirred for 2 h at room temperature. Then a saturated solution of NH_4_Cl (20 mL) was added, and the aqueous phase was extracted with CH_2_Cl_2_ (3 × 20 mL). The combined organic phases were washed with NH_4_Cl_sat._ (60 mL), water (60 mL), and brine (60 mL), dried (MgSO_4_) and filtered, and the solvent was removed under reduced pressure. The resulting residue was purified by column chromatography (30% ether/70% petroleum ether) to give a solid that was crystalized from ether to give compound **1b** as a white solid (0.196 g, 50%), m.p. 161–163 °C. ^1^H NMR (400 MHz, DMSO-*d_6_*): δ = 3.95 (s, 3H, OMe), 8.87 (d, *J* = 2.0 Hz, 1H, HetArH), 8.97 (d, *J* = 2.0 Hz, 1H, HetArH) ppm. ^13^C NMR (100.6 MHz, DMSO-*d*_6_): δ = 53.3 (OMe), 115.0 (C), 131.0 (C), 145.0 (CH), 145.2 (CH), 146.0 (C), 153.5 (C), 160.5 (C=O) ppm. HRMS (EI): [M]^+^ calculated for C_8_H_5_^79^BrN_2_O_2_S: 271.9255, found: 271.9254; for C_8_H_5_^81^BrN_2_O_2_S: 273.9235, found: 273.9243.

#### 3.1.2. General Procedure for the Synthesis of Diarylamines Using Reaction Conditions A

To a dried Schlenk tube with dry 1,4-dioxane (2–3 mL), compound **1a** (1 equiv.), (hetero)arylhalide (1.1 equiv.), Pd(OAc)_2_ (10 mol.%), Xantphos (12 mol.%), and Cs_2_CO_3_ (2.8 equiv.) were added under argon. The reaction was stirred at 120 °C for 2–5 h. After cooling, the reaction mixture was passed through a pad of silica-gel covered with celite, using ether or AcOEt (50 mL). The removal of the solvent gave a solid that was washed with ether to isolate the product or the crude. The latter was submitted to column chromatography using a solvent gradient, increasing 10% each time, from 10/90 of ether/petroleum ether until the isolation of the product, unless stated.

#### 3.1.3. General Procedure for the Synthesis of Diarylamines Using Reaction Conditions B

To a dried Schlenk tube with dry toluene (2–3 mL), compound **1b** (1 equiv.), (hetero)arylamine (1.1 equiv.), Pd(OAc)_2_ (6 mol.%), *rac*-BINAP (8 mol.%), and Cs_2_CO_3_ (2 equiv.) were added, under argon. The reaction was stirred at 100 °C for 2–6 h, and after cooling, the reaction mixture was passed through a pad of silica-gel covered with celite, using ether or AcOEt (50 mL). The removal of the solvent gave a solid that was washed with ether to isolate the product or the crude. The latter was submitted to column chromatography using a gradient of solvents, increasing 10% each time, from 10/90 of ether/petroleum ether until the isolation of the product, unless stated.

##### Methyl 7-(Phenylamino)thieno[2,3-b]pyrazine-6-carboxylate (**2a**)

From compound **1a** (0.380 mmol, 80.0 mg), bromobenzene (0.420 mmol, 44.3 μL), following reaction conditions A and heating for 5 h, compound **2a** was obtained as a yellow solid after dry flash column chromatography using 40% ether/petroleum ether (55.0 mg, 50%), m.p. 154–156 °C. ^1^H NMR (400 MHz, CDCl_3_): δ = 3.96 (s, 3H, OMe), 7.10–7.13 (m, 3H, ArH), 7.27–7.31 (m, 2H, ArH), 8.51 (d, *J* = 2.0 Hz, 1H, HetArH), 8.58 (d, *J* = 2.0 Hz, 1H, HetArH), 8.87 (broad s, 1H, NH) ppm. ^13^C NMR (100.6 MHz, CDCl_3_): δ = 52.3 (OMe), 107.0 (C), 121.5 (2 × CH), 124.0 (CH), 129.0 (2 × CH), 140.6 (CH), 141.0 (C), 142.0 (C), 143.1 (C), 143.5 (CH), 155.5 (C), 165.2 (C=O). HRMS (ESI): [M + H]^+^ calculated for C_14_H_12_N_3_O_2_S: 286.0645, found: 286.0649.

##### Methyl 7-[(2-Methoxyphenyl)amino]thieno[2,3-*b*]pyrazine-6-carboxylate (**2b**)

From compound **1b** (0.370 mmol, 100.0 mg), *o*-anisidine (0.400 mmol, 45.4 μL), following reaction conditions B and heating for 4 h, compound **2b** was obtained as a pale yellow solid (75.0 mg, 65%), m.p. 186–188 °C. ^1^H NMR (400 MHz, DMSO-*d*_6_): δ = 3.78 (s, 3H, 2′-OMe), 3.87 (s, 3H, OMe), 6.76–6.82 (m, 1H, ArH), 7.01–7.03 (m, 2H, ArH), 7.06 (broad d, *J* = 7.6 Hz, 1H, ArH), 8.64 (d, *J* = 2.4 Hz, 1H, HetArH), 8.75 (d, *J* = 2.4 Hz, 1H, HetArH), 8.77 (broad s, 1H, NH) ppm. ^13^C NMR (100.6 MHz, DMSO-*d*_6_): δ = 52.4 (OMe), 56.0 (2′-OMe), 105.5 (C), 111.0 (CH), 120.0 (CH), 121.0 (CH), 123.5 (CH), 130.0 (C), 141.4 (CH), 142.0 (C), 142.2 (C), 144.5 (CH), 150.2 (2′-C), 154.0 (C), 164.2 (C=O) ppm. HRMS (ESI): [M + H]^+^ calculated for C_15_H_14_N_3_O_3_S: 316.0750, found: 316.0751.

##### Methyl 7-[(3-Methoxyphenyl)amino]thieno[2,3-*b*]pyrazine-6-carboxylate (**2c**)

From compound **1b** (0.370 mmol, 100 mg), *m*-anisidine (0.400 mmol, 45.4 μL), following reaction conditions B and heating for 2 h, compound **2c** was obtained as a pale yellow solid (92.0 mg, 80%), m.p. 171–173 °C. ^1^H NMR (400 MHz, DMSO-*d*_6_): δ = 3.65 (s, 3H, 3′-OMe), 3.77 (s, 3H, OMe), 6.48 (apparent dd, *J* = 8.0 and 2.0 Hz, 1H, ArH), 6.56 (broad d, *J* = 8.0 Hz, 1H, ArH), 6.60 (apparent t, *J* = 2.0 Hz, 1H, 2′-H), 7.05 (apparent t, *J* = 8.0 Hz, 1H, 5′-H), 8.66 (d, *J* = 2.4 Hz, 1H, HetArH), 8.72 (d, *J* = 2.4 Hz, 1H, HetArH), 8.93 (broad s, 1H, NH) ppm. ^13^C NMR (100.6 MHz, DMSO-*d*_6_): δ = 52.4 (OMe), 55.0 (3′-OMe), 105.4 (2′-CH), 108.0 (CH), 109.1 (C), 112.0 (CH), 129.0 (5′-CH), 141.0 (C), 142.0 (CH), 142.3 (C), 143.2 (C), 144.5 (CH), 154.0 (C), 159.5 (3′-C), 163.4 (C=O) ppm. HRMS (ESI): [M + H]^+^ calculated for C_15_H_14_N_3_O_3_S: 316.0750, found: 316.0754.

##### Methyl 7-[(4-Methoxyphenyl)amino]thieno[2,3-*b*]pyrazine-6-carboxylate (**2d**)

From compound **1b** (0.370 mmol, 100.0 mg), *p*-anisidine (0.400 mmol, 49.6 mg), following reaction conditions B and heating for 4 h, compound **2d** was obtained as a pale yellow solid (87.0 mg, 75%), m.p. 147–149 °C. ^1^H NMR (400 MHz, DMSO-*d*_6_): δ = 3.72 (s, 3H, 4′-OMe), 3.83 (s, 3H, OMe), 6.81 (d, *J* = 8.8 Hz, 2H, 3′ and 5′-H), 7.05 (d, *J* = 8.8 Hz, 2H, 2′ and 6′-H), 8.60 (d, *J* = 2.4 Hz, 1H, HetArH), 8.71 (d, *J* = 2.4 Hz, 1H, HetArH), 8.88 (broad s, 1H, NH) ppm. ^13^C NMR (100.6 MHz, DMSO-*d*_6_): δ = 52.2 (OMe), 55.2 (4′-OMe), 105.0 (C), 114.0 (3′ and 5′-CH), 123.0 (2′ and 6′-CH), 134.4 (C), 141.3 (CH), 142.0 (C), 143.0 (C), 144.3 (CH), 154.3 (C), 155.5 (4′-C), 164.0 (C=O) ppm. HRMS (ESI): [M + H]^+^ calculated for C_15_H_14_N_3_O_3_S: 316.0750, found: 316.0753.

##### Methyl 7-[(2,4-Dimethoxyphenyl)amino]thieno[2,3-*b*]pyrazine-6-carboxylate (**2e**)

From compound **1b** (0.220 mmol, 60.0 mg), 2,4-dimethoxyaniline (0.240 mmol, 34.4 µL), following reaction conditions B and heating for 2 h, compound **2e** was obtained as a red solid (76.0 mg, quantitative yield), m.p. 159–161 °C. ^1^H NMR (400 MHz, CDCl_3_): δ = 3.78 (s, 3H, 2′-OMe), 3.83 (s, 3H, 4′-OMe), 3.95 (s, 3H, OMe), 6.41 (dd, *J* = 8.4 and 2.4 Hz, 1H, 5′-H), 6.52 (d, *J* = 2.4 Hz, 1H, 3′-H), 7.05 (d, *J* = 8.4 Hz, 1H, 6′-H), 8.46 (d, *J* = 2.4 Hz, 1H, HetArH), 8.53 (d, *J* = 2.4 Hz, 1H, HetArH), 8.79 (broad s, 1H, NH) ppm. ^13^C NMR (100.6 MHz, CDCl_3_): δ = 52.1 (OMe), 55.5 (4′-OMe), 55.6 (2′-OMe), 99.0 (3′-CH), 103.3 (5′-CH), 103.5 (C), 123.0 (C), 124.0 (6′-CH), 140.4 (CH), 142.2 (C), 143.3 (CH), 144.2 (C), 153.0 (2′-C), 155.5 (C), 157.4 (4′-C), 165.4 (C=O) ppm. HRMS (ESI): [M + H]^+^ calculated for C_16_H_16_N_3_O_4_S: 346.0856, found: 346.0856.

##### Methyl 7-[(3,4-Dimethoxyphenyl)amino]thieno[2,3-*b*]pyrazine-6-carboxylate (**2f**)

From compound **1b** (0.220 mmol, 60.0 mg), 3,4-dimethoxyaniline (0.240 mmol, 37.0 mg) following the reaction conditions B and heating for 2h, compound 2f was obtained as a red solid (65.0 mg, 86%), m.p. 167–169 °C. ^1^H NMR (400 MHz, CDCl_3_): δ = 3.79 (s, 3H, 3′-OMe), 3.89 (s, 3H, 4′-OMe), 3.96 (s, 3H, OMe), 6.68 (dd, *J* = 8.8 and 2.4 Hz, 1H, 6′-H), 6.74 (d, *J* = 2.4 Hz, 1H, 2′-H), 6.79 (d, *J* = 8.8 Hz, 1H, 5′-H), 8.49 (d, *J* = 2.0 Hz, 1H, HetArH), 8.56 (d, *J* = 2.0 Hz, 1H, HetArH), 8.92 (broad s, 1H, NH) ppm. ^13^C NMR (100.6 MHz, CDCl_3_): δ = 52.2 (OMe), 55.9 (3′-OMe), 56.1 (4′-OMe), 105.0 (C), 107.4 (2′-CH), 111.1 (5′-CH), 114.4 (6′-CH), 134.3 (C), 140.5 (CH), 142.0 (C), 143.4 (CH), 144.0 (C), 146.1 (4′-C), 149.0 (3′-C), 156.0 (C), 165.5 (C=O) ppm. HRMS (ESI): [M + H]^+^ calculated for C_16_H_16_N_3_O_4_S: 346.0856, found: 346.0856.

##### Methyl 7-[(3,5-Dimethoxyphenyl)amino]thieno[2,3-*b*]pyrazine-6-carboxylate (**2g**)

From compound **1b** (0.200 mmol, 55.0 mg), 3,5-dimethoxyaniline (0.220 mmol, 33.9 mg), following reaction conditions B and heating for 2 h, compound **2g** was obtained as a yellow solid (56.0 mg, 80%), m.p. 196–198 °C. ^1^H NMR (400 MHz, CDCl_3_): δ = 3.74 (s, 6H, 3′ and 5′-OMe), 3.96 (s, 3H, OMe), 6.22-6.26 (m, 3H, 4’, 2’ and 6′-H), 8.56-8.60 (m, 2H, HetArH), 8.78 (broad s, 1H, NH) ppm. ^13^C NMR (100.6 MHz, CDCl_3_): δ = 52.4 (OMe), 55.3 (3′ and 5′-OMe), 96.0 (4′-CH), 100.0 (2′ and 6′-CH), 108.4 (C), 141.0 (CH), 142.1 (C), 142.7 (C), 143.0 (C), 144.0 (CH), 155.4 (C), 161.0 (3′ and 5′-C), 165.1 (C=O). HRMS (ESI): [M + H]^+^ calculated for C_16_H_16_N_3_O_4_S: 346.0856, found: 346.0858.

##### Methyl 7-[(3,4,5-Trimethoxyphenyl)amino]thieno[2,3-*b*]pyrazine-6-carboxylate (**2h**)

From compound **1b** (0.220 mmol, 60.0 mg), 3,4-dimethoxyaniline (0.240 mmol, 44.3 mg), following reaction conditions B and heating for 2 h, compound **2h** was obtained as a dark orange solid (85.0 mg, quantitative yield), m.p. 194–196 °C. ^1^H NMR (400 MHz, DMSO-*d*_6_): δ = 3.62 (s, 3H, 4′-OMe), 3.63 (s, 6H, 3′ and 5′-OMe), 3.85 (s, 3H, OMe), 6.43 (s, 2H, 2′ and 6′-H), 8.69 (d, *J* = 2.0 Hz, 1H, HetArH), 8.75 (d, *J* = 2.0 Hz, 1H, HetArH), 8.93 (broad s, 1H, NH) ppm. ^13^C NMR (100.6 MHz, CDCl_3_): δ = 52.4 (OMe), 55.7 (3′ and 5′-OMe), 60.1 (4′-OMe), 98.4 (2′ and 6′-CH), 107.0 (C), 133.2 (4′-C), 137.4 (C), 141.4 (CH), 141.5 (C), 142.0 (C), 144.4 (CH), 153.0 (3′ and 5′-C), 154.2 (C), 164.0 (C=O) ppm. HRMS (ESI): [M + H]^+^ calculated for C_17_H_18_N_3_O_5_S: 376.0962, found: 376.0964.

##### Methyl 7-[(2-Fluorophenyl)amino]thieno[2,3-*b*]pyrazine-6-carboxylate (**2i**)

From compound **1b** (0.240 mmol, 65.0 mg), 2-fluoroaniline (0.260 mmol, 25.3 μL), following reaction conditions B and heating for 2 h, compound **2i** was obtained as a pale yellow solid after dry flash column chromatography till 10% ether/petroleum ether (59.0 mg, 82%), m.p. 137–139 °C. ^1^H NMR (400 MHz, CDCl_3_): δ = 3.97 (s, 3H, OMe), 7.03–7.13 (m, 3H), 7.19 (broad apparent t, *J* = 8.0 Hz, 1H, 5′-H), 8.49 (d, *J* = 2.0 Hz, 1H, HetArH), 8.58 (d, *J* = 2.0 Hz, 1H, HetArH), 8.81 (broad s, 1H, NH) ppm. ^13^C NMR (100.6 MHz, CDCl_3_): δ = 52.3 (OMe), 106.5 (C), 115.5 (d, *J* = 20.0 Hz, 3′-CH), 123.6 (d, *J* = 4.0 Hz, CH), 123.8 (d, *J* = 1.0 Hz, 5′-CH), 124.7 (d, *J* = 7.0 Hz, CH), 128.9 (d, *J* = 13.0 Hz, 1′-C), 141.0 (CH), 142.0 (C), 143.0 (C), 144.0 (CH), 155.3 (C), 155.5 (d, *J* = 245.0 Hz, C-F), 165.2 (C=O) ppm. ^19^F NMR (376.48 MHz, CDCl_3_): δ = −125.5 (s). HRMS (ESI): [M + H]^+^ calculated for C_14_H_11_FN_3_O_2_S: 304.0551, found: 304.0555.

##### Methyl 7-[(3-Fluorophenyl)amino]thieno[2,3-*b*]pyrazine-6-carboxylate (**2j**)

From compound **1b** (0.220 mmol, 60.0 mg), 3-fluoroaniline (0.240 mmol, 23.2 μL), following reaction conditions B and heating for 2 h, compound **2j** was obtained as a yellow solid (59.0 mg, 89%), m.p. 170–172 °C. ^1^H NMR (400 MHz, CDCl_3_): δ = 3.97 (s, 3H, OMe), 6.76–6.82 (m, 2H, ArH), 6.85–6.89 (m, 1H, 6′-H), 7.20–7.25 (m, 1H, 5′-H), 8.56 (d, *J* = 2.0 Hz, 1H, HetArH), 8.61 (d, *J* = 2.0 Hz, 1H, HetArH), 8.84 (broad s, 1H, NH) ppm. ^13^C NMR (100.6 MHz, CDCl_3_): δ = 52.4 (OMe), 108.2 (d, *J* = 25.0 Hz, CH), 109.0 (C), 110.1 (d, *J* = 22.0 Hz, CH), 116.5 (d, *J* = 2.0 Hz, 6′-CH), 129.7 (d, *J* = 9.0 Hz, 5′-CH), 141.0 (CH), 142.0 (C), 142.2 (C), 142.8 (d, *J* = 10.0 Hz, 1′-C), 144.0 (CH), 155.4 (C), 163.0 (d, *J* = 245.0 Hz, C-F), 165.1 (C=O) ppm. ^19^F NMR (376.48 MHZ, CDCl_3_): δ = −112.7 (s). HRMS (ESI): [M + H]^+^ calculated for C_14_H_11_FN_3_O_2_S: 304.0551, found: 304.0555.

##### Methyl 7-[(4-Fluorophenyl)amino]thieno[2,3-*b*]pyrazine-6-carboxylate (**2k**)

From compound **1b** (0.400 mmol, 110.0 mg), 4-fluoroaniline (0.440 mmol, 42.0 µL), following reaction conditions B and heating for 2 h, compound **2k** was obtained as an orange solid (86.0 mg, 70%), m.p. 171–173 °C. ^1^H NMR (400 MHz, DMSO-*d*_6_): δ = 3.83 (s, 3H, OMe), 7.02–7.07 (m, 2H, 3′ and 5′-H), 7.08–7.12 (m, 2H, 2′ and 6′-H), 8.64 (d, *J* = 2.4 Hz, 1H, HetArH), 8.74 (d, *J* = 2.4 Hz, 1H, HetArH), 8.97 (broad s, 1H, NH) ppm. ^13^C NMR (100.6 MHz, DMSO-*d*_6_): δ = 52.4 (OMe), 107.3 (C), 114.8 (d, *J* = 22.0 Hz, 3′ and 5′-CH), 122.2 (d, *J* = 9.0 Hz, 2′ and 6′-CH), 138.1 (C), 138.12 (C), 141.5 (CH), 142.0 (C), 144.4 (CH), 154.2 (C), 158.1 (d, *J* = 238.4 Hz, C-F), 163.5 (C=O) ppm. ^19^F NMR (376.48 MHz, DMSO-*d_6_*): -121.1 (s). HRMS (ESI): [M + H]^+^ calculated for C_14_H_11_FN_3_O_2_S: 304.0551, found: 304.0556.

##### Methyl 7-[(Pyridin-3-yl)amino]thieno[2,3-*b*]pyrazine-6-carboxylate (**2l**)

From compound **1a** (0.380 mmol, 80.0 mg), 3-bromopyridine (0.420 mmol, 40.5 μL), following reaction condition A and heating for 2 h, compound **2l** was obtained as an orange solid after dry flash column chromatography till 80% ethyl acetate/petroleum ether (60.0 mg, 55%), m.p. 150–152 °C. ^1^H NMR (400 MHz, CDCl_3_): δ = 3.98 (s, 3H, OMe), 7.23 (dd, *J* = 8.4 and 4.8 Hz, 1H, 5′-H), 7.42–7.45 (m, 1H, 4’-H), 8.35 (dd, *J* = 4.8 and 1.2 Hz, 1H, 6′-H), 8.46-8.47 (m, 1H, 2′-H), 8.50 (d, *J* = 2.0 Hz, 1H, HetArH), 8.60 (d, *J* = 2.0 Hz, 1H, HetArH), 8.92 (broad s, 1H, NH) ppm. ^13^C NMR (100.6 MHz, CDCl_3_): δ = 52.4 (OMe), 108.0 (C), 123.0 (5′-CH), 128.4 4′-CH), 137.3 (C), 141.0 (CH), 141.5 (C), 142.2 (C), 143.6 (2′-CH), 144.0 (CH), 145.0 (6′-CH), 155.5 (C), 165.3 (C=O) ppm. HRMS (ESI): [M + H]^+^ calculated for C_13_H_11_N_4_O_2_S: 287.0597, found: 287.0592.

##### Methyl 7-(1*H*-pyrrol-1-yl)thieno[2,3-*b*]pyrazine-6-carboxylate (**2m**)

From compound **1b** (0.380 mmol, 80.0 mg), 1*H*-pyrrol (0.420 mmol, 40.5 μL), following reaction conditions B and heating for 6 h, compound **2m** was obtained as an pale yellow solid after dry flash column chromatography till 30% ether/petroleum ether (54.0 mg, 71%), m.p. 166–168 °C. ^1^H NMR (400 MHz, CDCl_3_): δ = 3.93 (s, 3H, OMe), 6.45 (apparent t, *J* = 2.0 Hz, 2H, 2 × HetArH), 7.13 (apparent t, *J* = 2.0 Hz, 2H, 2 × HetArH), 8.70 (d, *J* = 2.0 Hz, 1H, HetArH), 8.79 (d, *J* = 2.0 Hz, 1H, HetArH) ppm. ^13^C NMR (100.6 MHz, CDCl_3_): δ = 53.0 (OMe), 110.0 (2 × CH), 123.0 (2 × CH), 126.0 (C), 136.0 (C), 143.3 (CH), 144.0 (CH), 144.3 (C), 154.3 (C), 161.1 (C=O) ppm. HRMS (ESI): [M + H]^+^ calculated for C_12_H_10_N_3_O_2_S: 260.0488, found: 260.0486.

##### Methyl 7-[(4-Cyanophenyl)amino]thieno[2,3-*b*]pyrazine-6-carboxylate (**2n**)

From compound **1a** (0.380 mmol, 80.0 mg), 4-bromobenzonitrile (0.400 mmol, 76.5 mg), following reaction conditions A and heating 3.5 h, compound **2n** was obtained as a yellow solid (119 mg, quantitative yield), m.p. 214–216 °C. ^1^H NMR (400 MHz, CDCl_3_): δ = 3.99 (s, 3H, OMe), 7.11 (d, *J* = 8.8 Hz, 2H, 2’ and 6’-H), 7.54 (d, *J* = 8.8 Hz, 2H, 3’ and 5’-H), 8.60 (d, *J* = 2.0 Hz, 1H, HetArH), 8.66 (d, *J* = 2.0 Hz, 1H, HetArH), 8.90 (broad s, 1H, NH) ppm. ^13^C NMR (100.6 MHz, CDCl_3_): δ = 53.0 (OMe), 105.3 (C), 112.0 (C), 119.3 (C), 120.0 (2’ and 6’-CH), 133.0 (3’ and 5’-CH), 140.5 (C), 141.1 (CH), 142.0 (C), 144.0 (CH), 145.2 (C), 155.3 (C), 165.0 (C=O) ppm. HRMS (ESI): [M + H]^+^ calculated for C_15_H_11_N_4_O_2_S: 311.0597, found: 311.0598.

##### Methyl 7-[(2-Nitrophenyl)amino]thieno[2,3-*b*]pyrazine-6-carboxylate (**2o**)

From compound **1a** (0.450 mmol, 95.0 mg), 2-nitrobenzene (0.500 mmol, 101.0 mg), following reaction conditions A and heating 3 h, compound **2o** was obtained as a yellow solid after dry flash column chromatography till 100% ethyl acetate (90.0 mg, 60%), m.p. 225–227 °C. ^1^H NMR (400 MHz, CDCl_3_): δ = 4.01 (s, 3H, OMe), 6.96–7.04 (m, 2H, ArH), 7.35–7.40 (m, 1H, ArH), 8.20 (dd, *J* = 8.0 and 1.6 Hz, 1H, 3′-H), 8.61 (d, *J* = 2.0 Hz, 1H, HetArH), 8.67 (d, *J* = 2.0 Hz, 1H, HetArH), 10.65 (broad s, 1H, NH) ppm. ^13^C NMR (100.6 MHz, CDCl_3_): δ = 53.0 (OMe), 117.5 (C), 120.4 (CH), 121.0 (CH), 126.0 (3′-CH), 134.0 (CH), 136.4 (C), 137.5 (C), 138.4 (C), 141.4 (CH), 142.5 (C), 144.0 (CH), 155.0 (C), 163.4 (C=O). HRMS (ESI): [M + H]^+^ calculated for C_14_H_11_N_4_O_4_S: 331.0496, found: 331.0496.

### 3.2. In Vitro Antitumor Evaluation

#### 3.2.1. Cell Growth Inhibition Assay (SRB Assay)

The cell growth inhibition of compounds **2a**–**2o** DMSO/water solutions (3.9–250 µM) was evaluated against four human tumor cell lines (acquired from Leibniz-Institut DSMZ), namely: gastric adenocarcinoma (AGS), colorectal adenocarcinoma (CaCo-2), breast carcinoma (MCF7), and non-small-cell lung carcinoma (NCI-H460), as well as a non-tumor culture from African green monkey kidney (Vero). Each cell line was prepared in 96-well plates, at the required density (1.0 × 10^4^ cells/well) and incubated for 24 h to achieve cell attachment. The solutions of the compounds were applied and incubated for another 48 h. GI_50_ values (µM) corresponding to the compound concentration that inhibited 50% of cell growth were determined using the sulforhodamine B assay [24,25]. Two separate tests were carried out for each compound, in duplicate, with the effects expressed as mean values and standard deviation (SD). Ellipticine was used as a positive control.

#### 3.2.2. Flow Cytometric: Cell Cycle Analysis

AGS cells were seeded in six-well plates (4 × 10^5^ cells/well) and incubated with the compounds **2b**, **2f**, and **2g,** at their GI_50_ concentration for each sample, for 72 h. Cell cycle analysis was performed using propidium iodide (PI) staining and flow cytometry. This assay is established on the measurement of the DNA content of nuclei labeled with PI. Following this, the cells were staining according to the protocol PI/RNASE Solution (Immunostep, Spain, Salamanca). The harvested cells corresponding to 2 × 10^5^ to 1 × 10^6^ cells, were centrifuged for 5 min at 300*g*, and the supernatant was removed. The cells were fixed and added to 200 µL of 70% ethanol and left in the ethanol at 4 °C for 30 min. Following this, the cells were washed once in 2 mL phosphate-buffered saline (PBS) and centrifuged for 5 min at 300*g* and resuspended in 500 μL of PI solution (PI/RNASE) and incubated in the dark, at room temperature, for 15 min. Cell cycle phase distribution was evaluated using an Accuri C6 flow cytometer (BD Biosciences, San Jose, CA, USA). The DNA content of at least 20,000 cells was counted per sample, and the percentage of cells in different phases (G0/G1, S, and G2/M phases) of the cell cycle was evaluated using BD Accuri C6 software (BD Biosciences, San Jose, CA, USA) [26]. Ellipticine was used as a positive control at its GI_50_ concentration for AGS cell line.

#### 3.2.3. Flow Cytometric: Apoptosis Detection

AGS cells were seeded in six-well plates (4 × 10^5^ cells/well) and incubated with compounds **2b**, **2f**, and **2g** at their GI_50_ concentration for each sample, for 72 h. Apoptosis detection was performed using the Fluorescein Isothiocyanate (FITC) Annexin V Apoptosis Kit (BD Biosciences, San Jose, CA, USA) and flow cytometry. FITC Annexin V staining is used to determine the percentage of cells within a population that are actively undergoing apoptosis. The cells were washed twice with PBS and resuspended cells in 1× Binding Buffer (10 mM HEPES/NaOH, pH 7.4, 140 mM NaCl, 2.5 mM CaCl_2_) at a concentration of 1 × 10^6^ cells/mL. Following this, 100 μL of the solution (1 × 10^5^ cells) was transferred to a 5 mL culture tube and 5 μL of FITC Annexin V (BD Biosciences, San Jose, CA, USA) and 5 μL of PI (BD Biosciences, San Jose, CA, USA) were added to each tube, and they were incubated in the dark, at room temperature, for 15 min. Finally, 400 μL of 1× Binding Buffer was added to each tube. Next, 30,000 cells were acquired using an Accuri C6 flow cytometer (BD Biosciences, San Jose, CA, USA), and the percentage of cell distribution was evaluated with Accuri C6 software (BD Biosciences, San Jose, CA, USA) [27]. Ellipticine was used as a positive control at its GI_50_ concentration for AGS cell line.

#### 3.2.4. Statistical Analysis

Statistical analysis of data was performed using SPSS Statistics (version 23 for Windows; IBM Corp., Armonk, NY, USA). Statistical differences between groups were assessed by the chi-square test and differences with a *p* < 0.05 were considered significant.

## 4. Conclusions

A series of novel di(hetero)arylamines were synthesized by C–N Buchwald–Hartwig cross-coupling of either aminated or brominated thieno[2,3-*b*]pyrazines with (hetero)arylhalides or (hetero)arylamines, respectively, in good-to-excellent yields, using different reaction conditions, taking into account the electronic character of the substrates. The antitumor potential of the compounds obtained was evaluated against four human tumor cell lines (AGS, CaCo-2, MCF7, and NCI-H460). Despite the variety of the compounds, the results showed that the most promising ones were the mono- or dimethoxylated, **2b** (2-OMeC_6_H_4_), **2f** (3,5-diOMeC_6_H_3_), and **2g** (3,4-diOMeC_6_H_3_) which presented the lowest GI_50_ values and selectively inhibited the cell growth of the AGS and/or CaCo-2 cell lines among the human tumor cell lines tested. Moreover, compound **2h** (3,4,5-triOMeC_6_H_2_) and **2n** (4-CNC_6_H_4_) showed to be selective against CaCo-2 cell line presenting GI_50_ = 9.2 and 10.9 µM, respectively. The toxicity of the compounds was evaluated in a non-tumor cell line (Vero), and they did not show relevant toxicity at their GI_50_ concentrations (presenting high GI_50_ values). Effects on the cell cycle profile and induction of apoptosis were evaluated for compounds **2b**, **2f**, and **2g** in the AGS cell line. Nevertheless, even for the most active compound against this cell line (GI_50_ = 7.8 µM), it was observed that a huge number of dead cells gave rise to an atypical distribution on the cell cycle profile and that these cells were not apoptotic, which indicates that a different mechanism of action for the AGS cell growth inhibition is involved.

## Figures and Tables

**Table 1:** Synthesis of di(hetero)arylamines **2a**–**2o** by C–N Buchwald–Hartwig cross-coupling.

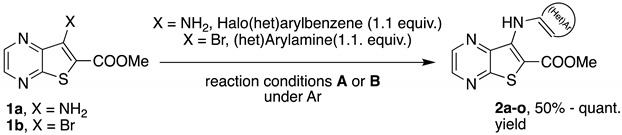					
Entry	Precursor, Conditions	Compounds 2	Entry	Precursor, Conditions	Compounds 2
1	**1a**, A	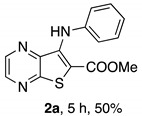	9	**1b**, B	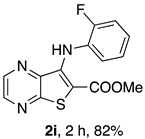
2	**1b**, B	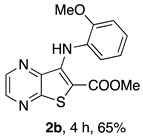	10	**1b**, B	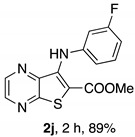
3	**1b**, B	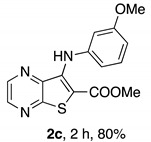	11	**1b**, B	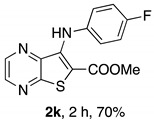
4	**1b**, B	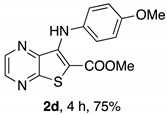	12	**1a**, A	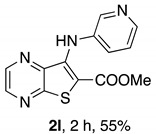
5	**1b**, B	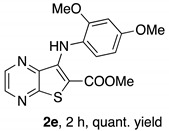	13	**1b**, B	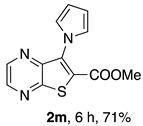
6	**1b**, B	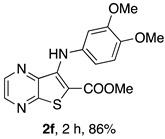	14	**1a**, A	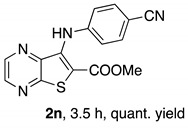
7	**1b**, B	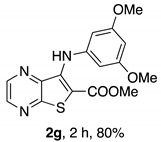	15	**1a**, A	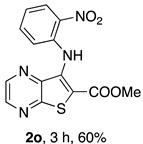
8	**1b**, B	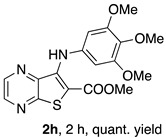			

Reaction conditions A: Pd(OAc)_2_ (10 mol.%), Xantphos (12 mol.%), Cs_2_CO_3_ (2.8 equiv.), 1,4-dioxane, 120 °C, 3–5 h. Reaction conditions B: Pd(OAc)_2_ (6 mol.%), *rac*-BINAP (8 mol.%), Cs_2_CO_3_ (2 equiv.), toluene, 100 °C, 2–6 h.

**Table 2:** GI_50_ concentrations of compounds **2a**–**2o** and the positive control Ellipticine in four human tumor cell lines and in a non-tumor cell ^1^.

GI_50_ (µM) ^1^					
Compounds 2	AGS	CaCo-2	MCF-7	NCI-H460	Vero
**2a**	95 ± 9	38 ± 2	97 ± 2	122 ± 9	120 ± 6
**2b**	**9.8 ± 0.2**	36 ± 2	127 ± 9	45 ± 1	**21.29 ± 0.03**
**2c**	98 ± 3	56 ± 1	160 ± 6	96 ± 1	94 ± 3
**2d**	59 ± 3	60 ± 2	136 ± 3	52.8 ± 0.3	127 ± 3
**2e**	34 ± 2	56 ± 2	140 ± 7	200 ± 3	138 ± 8
**2f**	**9.2 ± 0.2**	**8 ± 1**	87 ± 1	41 ± 3	**53 ± 3**
**2g**	**7.8 ± 0.2**	38 ± 4	182 ± 5	120 ± 10	**144 ± 10**
**2h**	33 ± 3	**9.2 ± 0.3**	105.7 ± 0.5	77 ± 6	**53 ± 5**
**2i**	48 ± 4	88.4 ± 0.2	128 ± 5	76 ± 1	117 ± 10
**2j**	86 ± 6	142 ± 11	96 ± 6	69 ± 7	149 ± 4
**2k**	118 ± 2	62 ± 4	160 ± 7	84 ± 2	149 ± 7
**2l**	63.3 ± 0.1	45 ± 3	116 ± 5	122 ± 11	110 ± 5
**2m**	88.6 ± 0.1	43.3 ± 0.3	128 ± 2	49 ± 2	140 ± 12
**2n**	39 ± 3	**10.9 ± 0.4**	103 ± 2	105 ± 8	**48 ± 4**
**2o**	55.0 ± 0.3	31.2 ± 0.2	103 ± 3	43 ± 2	92 ± 8
**Ellipticine (positive control)**	0.9 ± 0.1	0.8 ± 0.1	1.020 ± 0.004	1.01 ± 0.01	0.6 ± 0.1

^1^ GI_50_ values correspond to the compound concentration that causes 50% of cell growth inhibition. Results are expressed as mean values ± standard deviation.

## Data Availability

The data presented in this study are available in article and in the Supplementary Materials.

## Supplementary Materials

The following are available online, ^1^H, ^13^C, and ^19^F NMR spectra of the novel compounds **1b** and **2a**–**2o** are presented.

## Sample Availability

Not available.
