# Supplementary material for: Synthesis of Novel Methyl 7-[(Hetero)arylamino]thieno[2,3-b]pyrazine-6-carboxylates and Antitumor Activity Evaluation: Effects in Human Tumor Cells Growth, Cell Cycle Analysis, Apoptosis and Toxicity in Non-Tumor Cells
Source: Molecules. 2021 Aug 10;26(16):4823. doi: 10.3390/molecules26164823 (PMC8400120; doi:10.3390/molecules26164823)

Supplementary Material

Synthesis of Novel Methyl 7-[(Hetero)Arylamino]Thieno[2,3-*b*]Pyrazine-6-Carboxylates and Antitumor Activity Evaluation: Effects in Human Tumor Cells growth, Cell Cycle Analysis, Apoptosis and Toxicity in Non-tumor Cells

Juliana M. Rodrigues[1], Ricardo C. Calhelha[2], António Nogueira[2], Isabel C.F.R. Ferreira[2], Lillian Barros[2], and Maria-João R. P. Queiroz[1,*]

[1] Centro de Química- Universidade do Minho (CQUM), Campus de Gualtar 4710-057 Braga Portugal; id7018@alunos.uminho.pt (J.M.R.); mjrpq@quimica.uminho.pt (M.-J.R.P.Q.)
[2] Centro de Investigação de Montanha (CIMO) - Instituto Politécnico de Bragança, Campus de Santa Apolónia 5300-253 Bragança, Portugal; calhelha@ipb.pt (R.C.C.) ajmnogueira@ipb.pt (A.N.);iferreira@ipb.pt (I.C.F.R.F); lillian@ipb.pt (L.B.)
*Correspondence: mjrpq@quimica.uminho.pt (M.-J.R.P.Q.)

^{1}H and ^{13}C spectra of compound **1b**……………………………………………2
^{1}H and ^{13}C spectra of compound **2a**……………………………………………3
^{1}H and ^{13}C spectra of compound **2b**……………………………………………4
^{1}H and ^{13}C spectra of compound **2c**……………………………………………5
^{1}H and ^{13}C spectra of compound **2d**……………………………………………6
^{1}H and ^{13}C spectra of compound **2e**……………………………………………7
^{1}H and ^{13}C spectra of compound **2f**……………………………………………8
^{1}H and ^{13}C spectra of compound **2g**……………………………………………9
^{1}H and ^{13}C spectra of compound **2h**……………………………………………10
^{1}H, ^{13}C and ^{19}F spectra of compound **2i**……………………………………11
^{1}H, ^{13}C and ^{19}F spectra of compound **2j**……………………………………13
^{1}H, ^{13}C and ^{19}F spectra of compound **2k**……………………………………15
^{1}H and ^{13}C spectra of compound **2l**……………………………………………17
^{1}H and ^{13}C spectra of compound **2m**……………………………………………18
^{1}H and ^{13}C spectra of compound **2n**……………………………………………19
^{1}H and ^{13}C spectra of compound **2o**……………………………………………20

Methyl 7-bromothieno[2,3-*b*]pyrazine-6-carboxylate 1b

^{1}H NMR (DMSO-d_6, 400 MHz) of compound **1b**

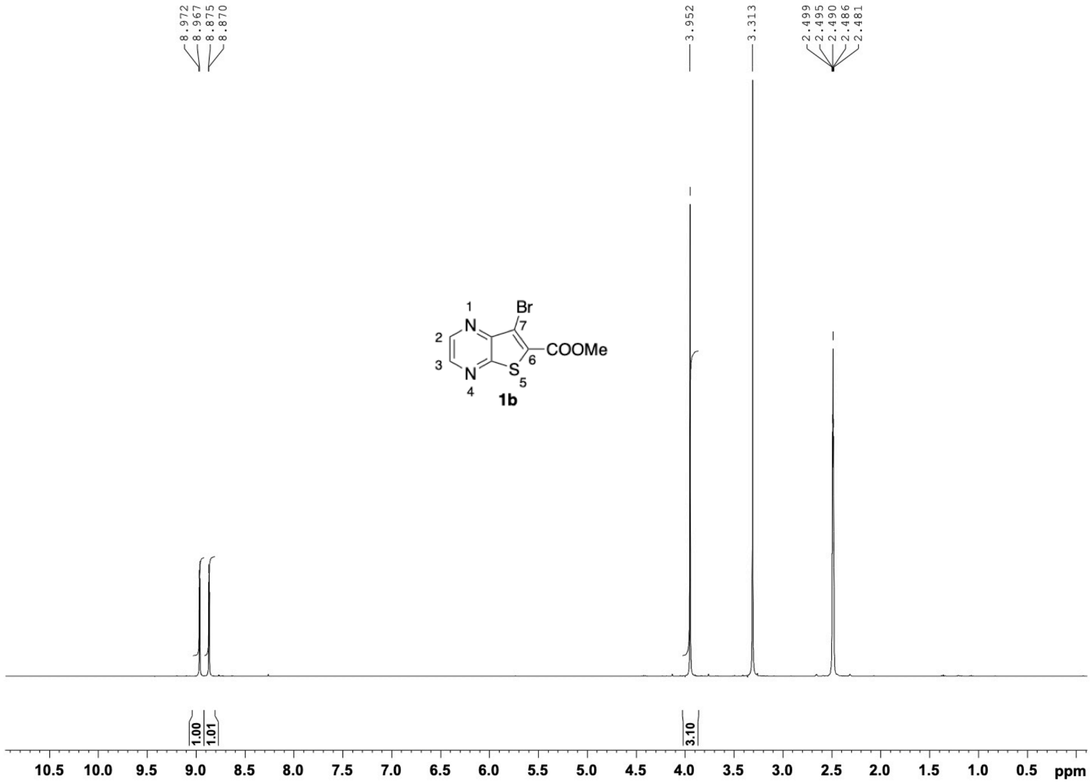

^{13}C NMR (DMSO-d_6, 100.6 MHz) of compound **1b**

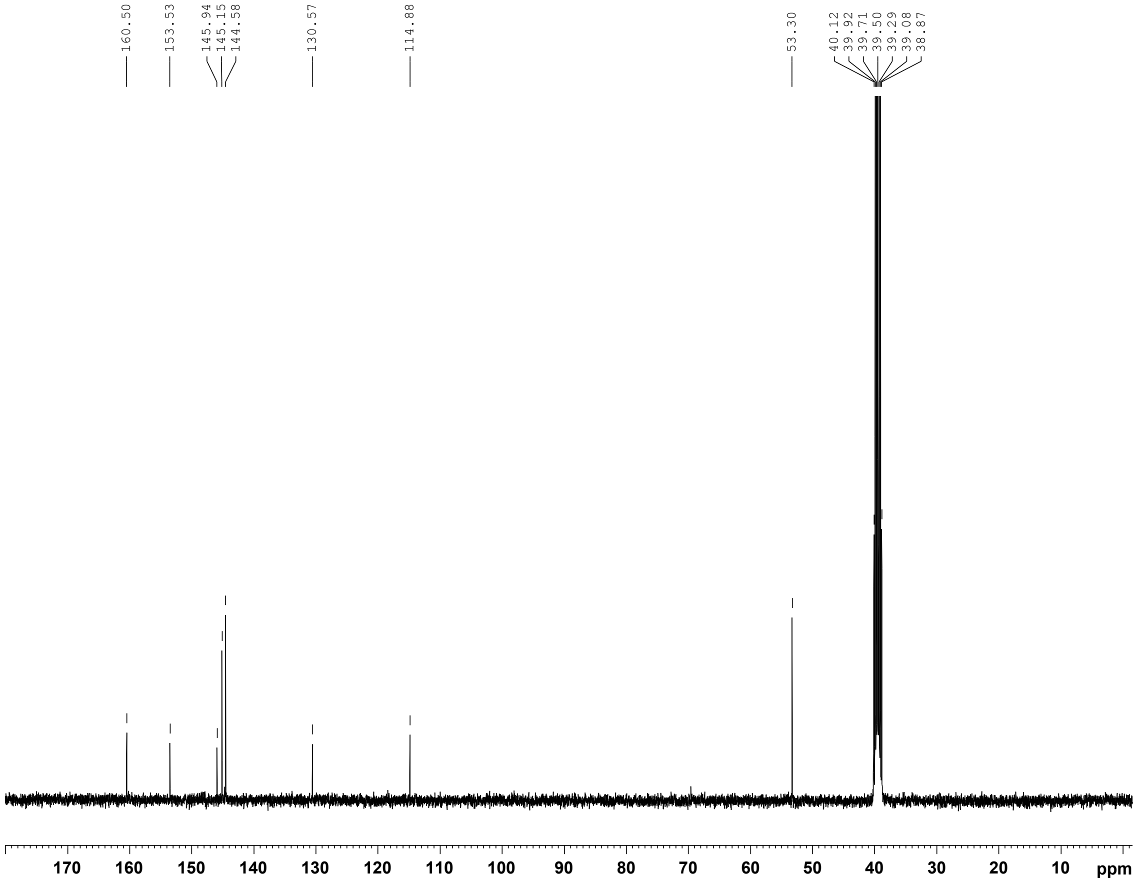

Methyl 7-(phenylamino)thieno[2,3-*b*]pyrazine-6-carboxylate 2a

^{1}H NMR ($CDCl_3$, 400 MHz) of compound **2a**

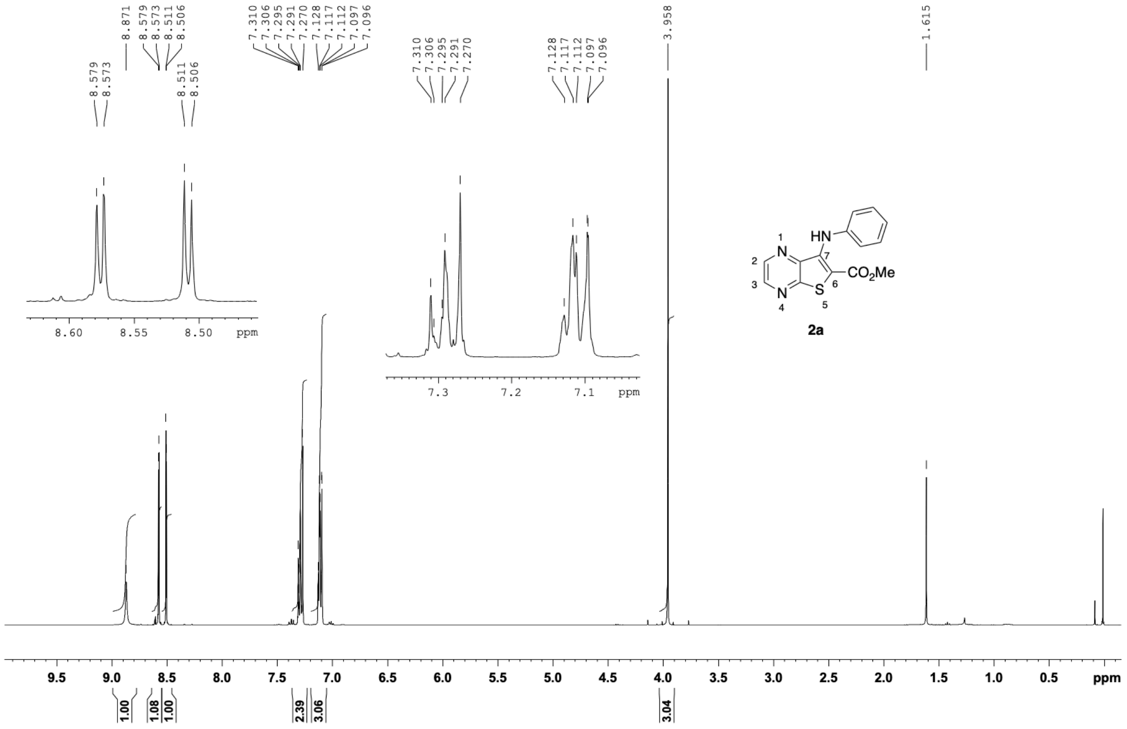

^{13}C NMR ($CDCl_3$, 100.6 MHz) of compound **2a**

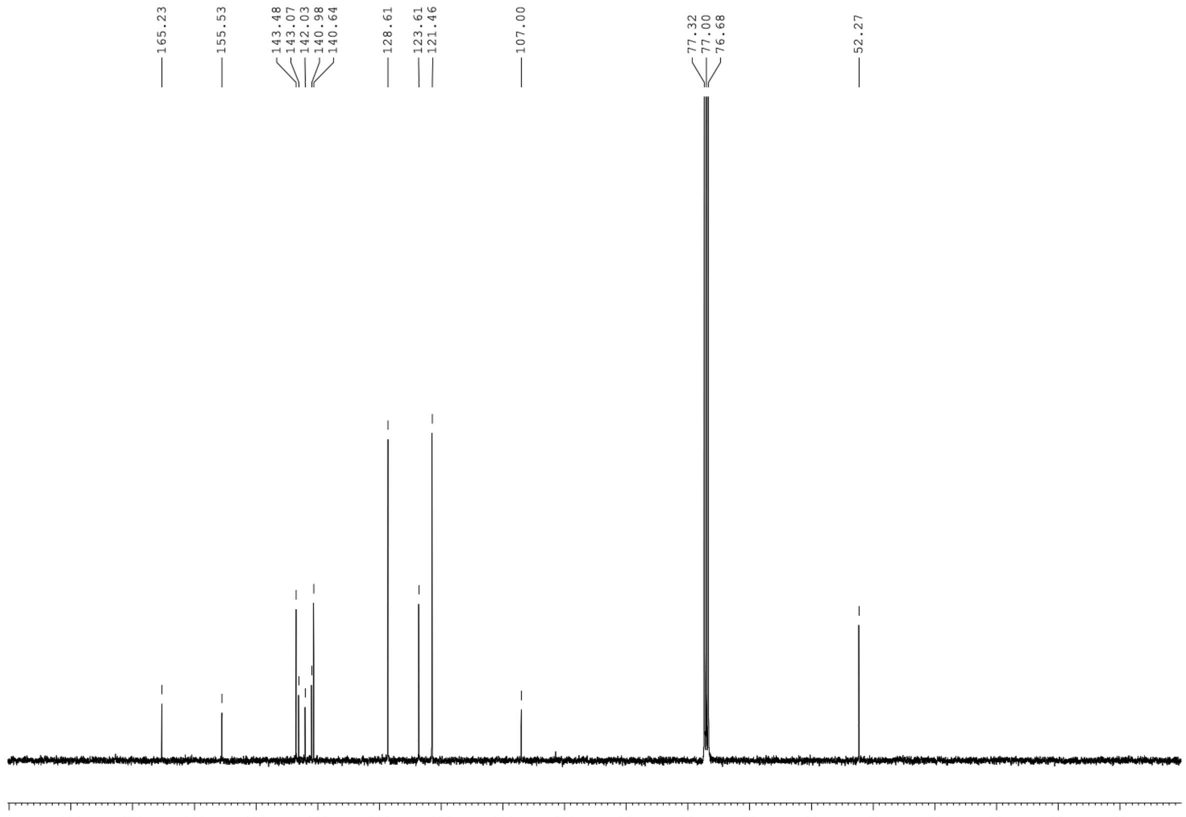

Methyl 7-[(2-methoxyphenyl)amino]thieno[2,3-*b*]pyrazine-6-carboxylate 2b

^{1}H NMR (DMSO-*d$_6$*, 400 MHz) of compound **2b**

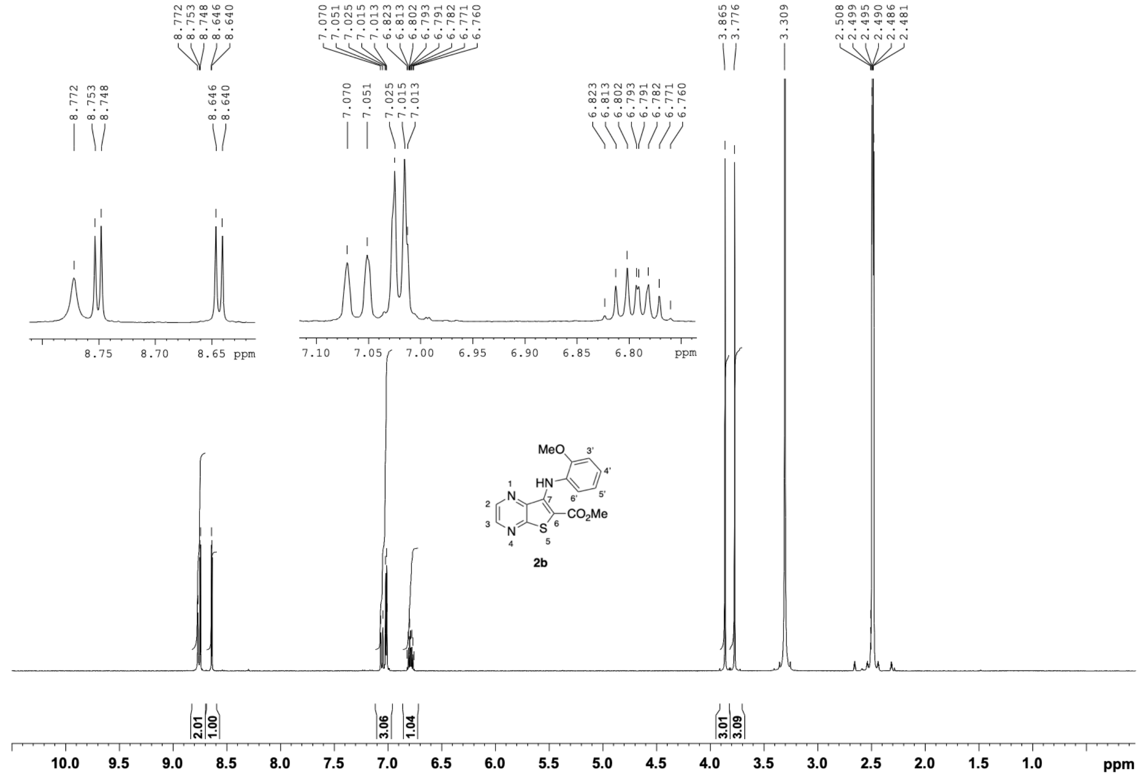

^{13}C NMR (DMSO-*d$_6$*, 100.6 MHz) of compound **2b**

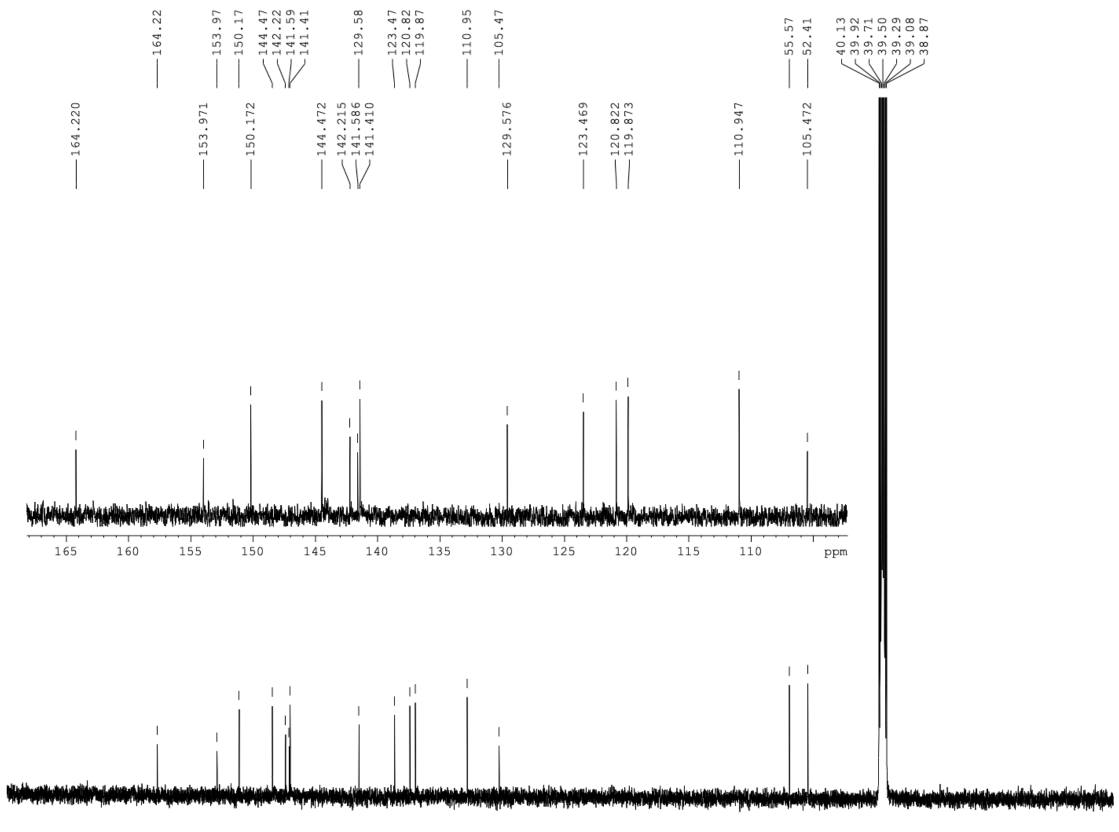

Methyl 7-[(3-methoxyphenyl)amino]thieno[2,3-*b*]pyrazine-6-carboxylate 2c

^{1}H NMR (DMSO-d_6, 400 MHz) of compound **2c**

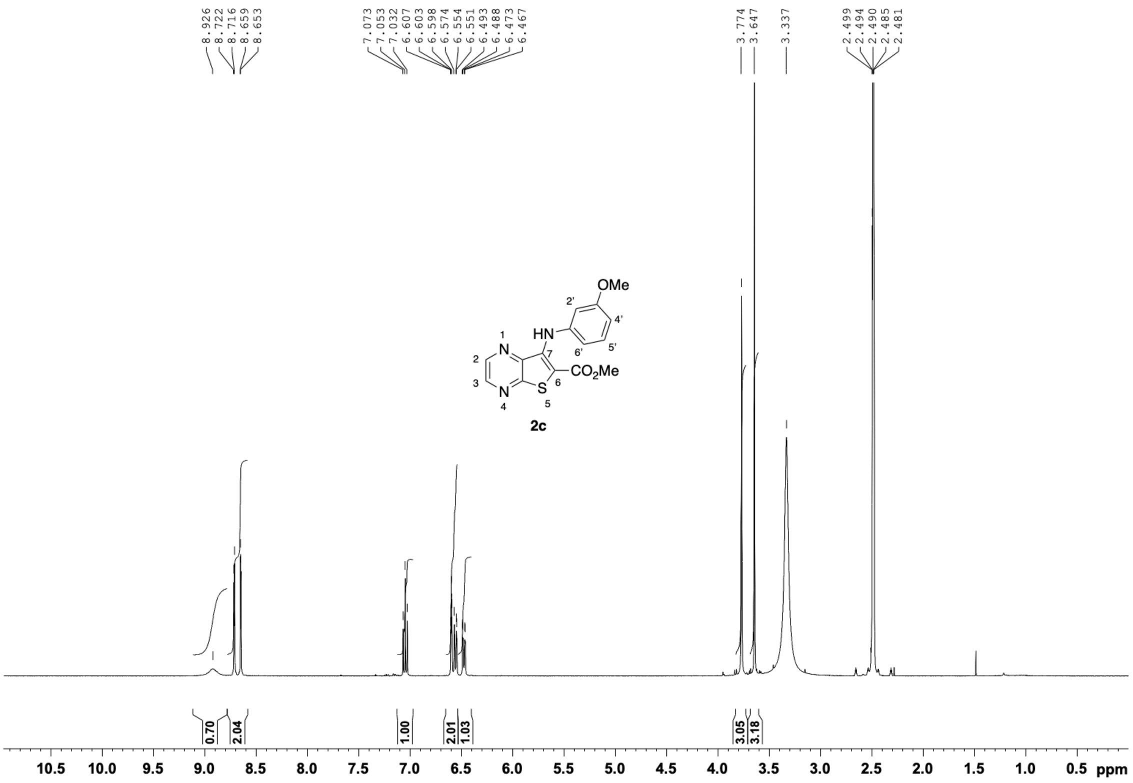

^{13}C NMR (DMSO-d_6, 100.6 MHz) of compound **2c**

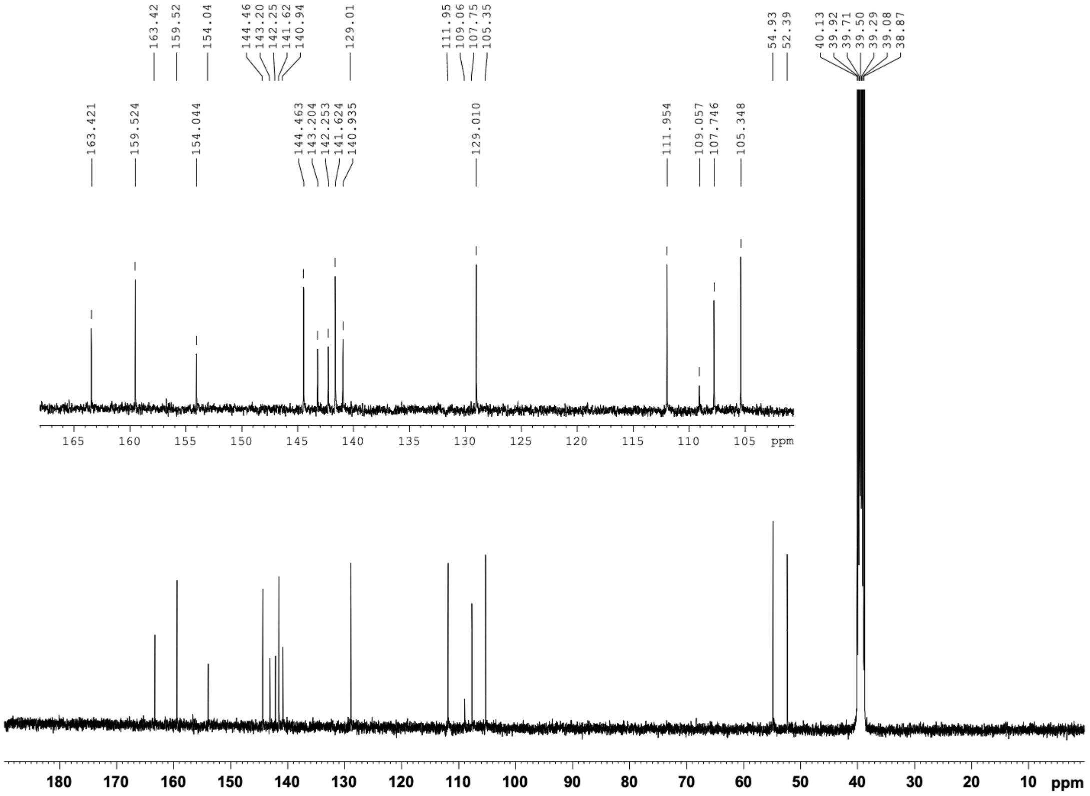

Methyl 7-[(4-methoxyphenyl)amino]thieno[2,3-*b*]pyrazine-6-carboxylate 2d

^{1}H NMR (DMSO-*d$_6$*, 400 MHz) of compound **2d**

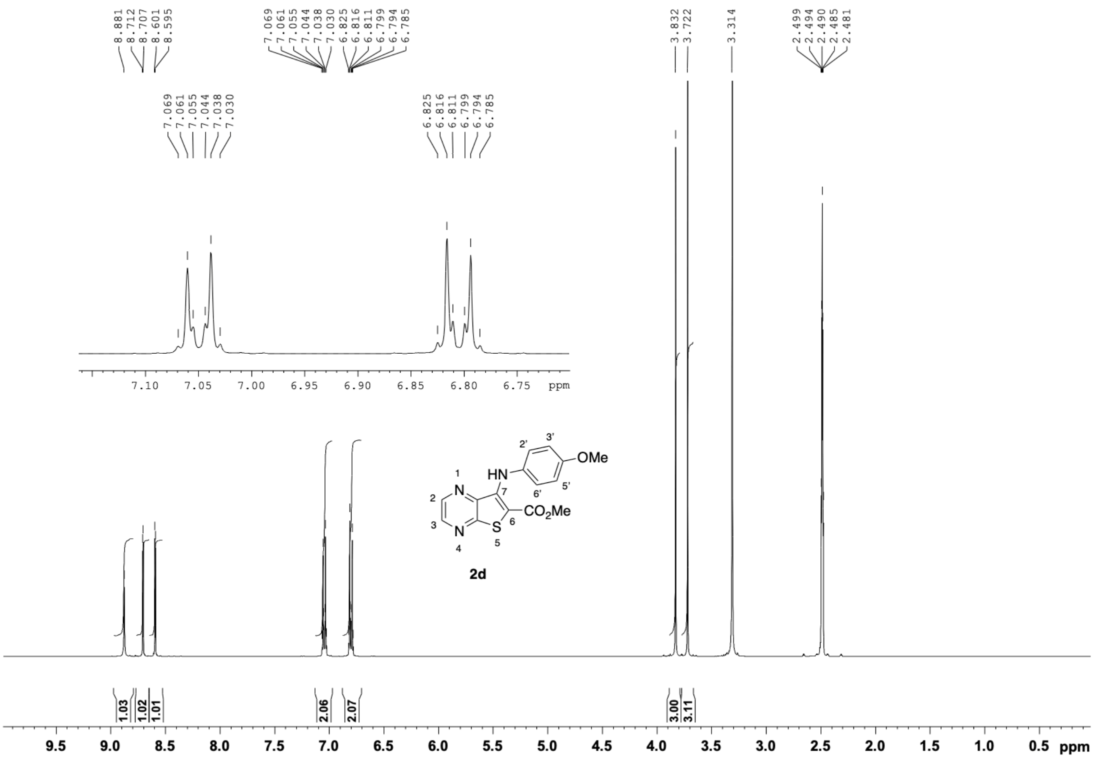

^{13}C NMR (DMSO-*d$_6$*, 100.6 MHz) of compound **2d**

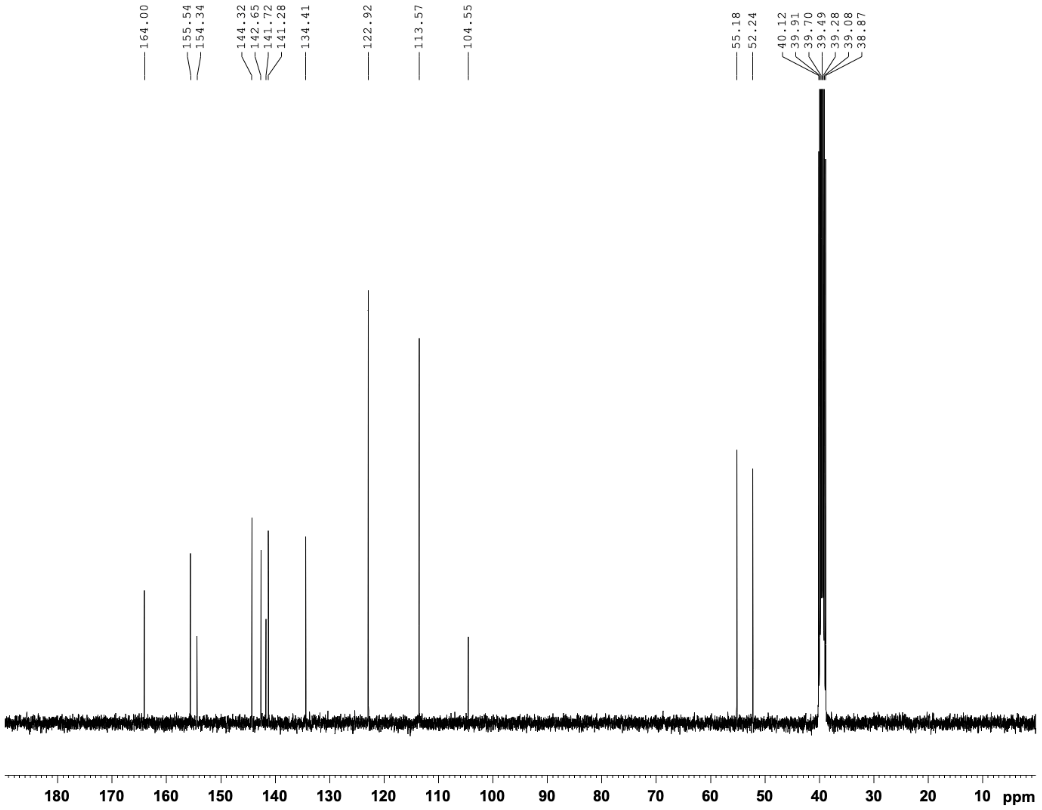

Methyl 7-[(2,4-dimethoxyphenyl)amino]thieno[2,3-*b*]pyrazine-6-carboxylate 2e

^{1}H NMR ($CDCl_3$, 400 MHz) of compound **2e**

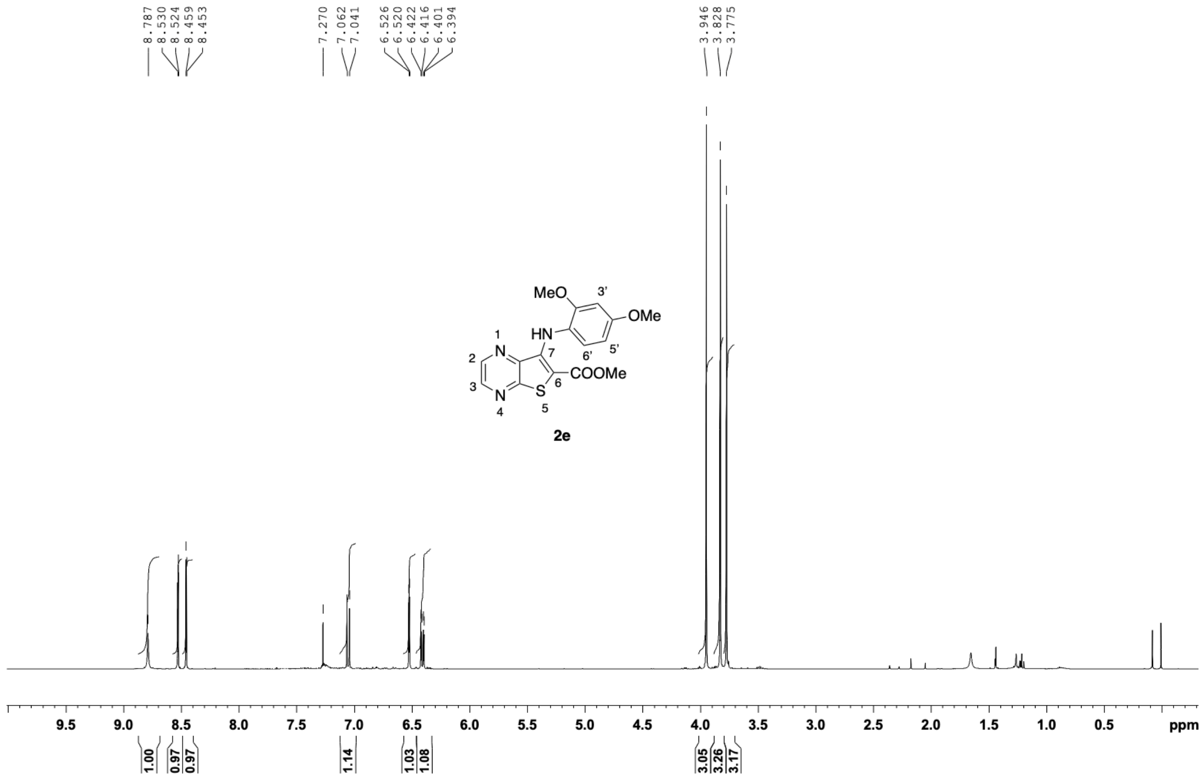

^{13}C NMR ($CDCl_3$, 100.6 MHz) of compound **2e**

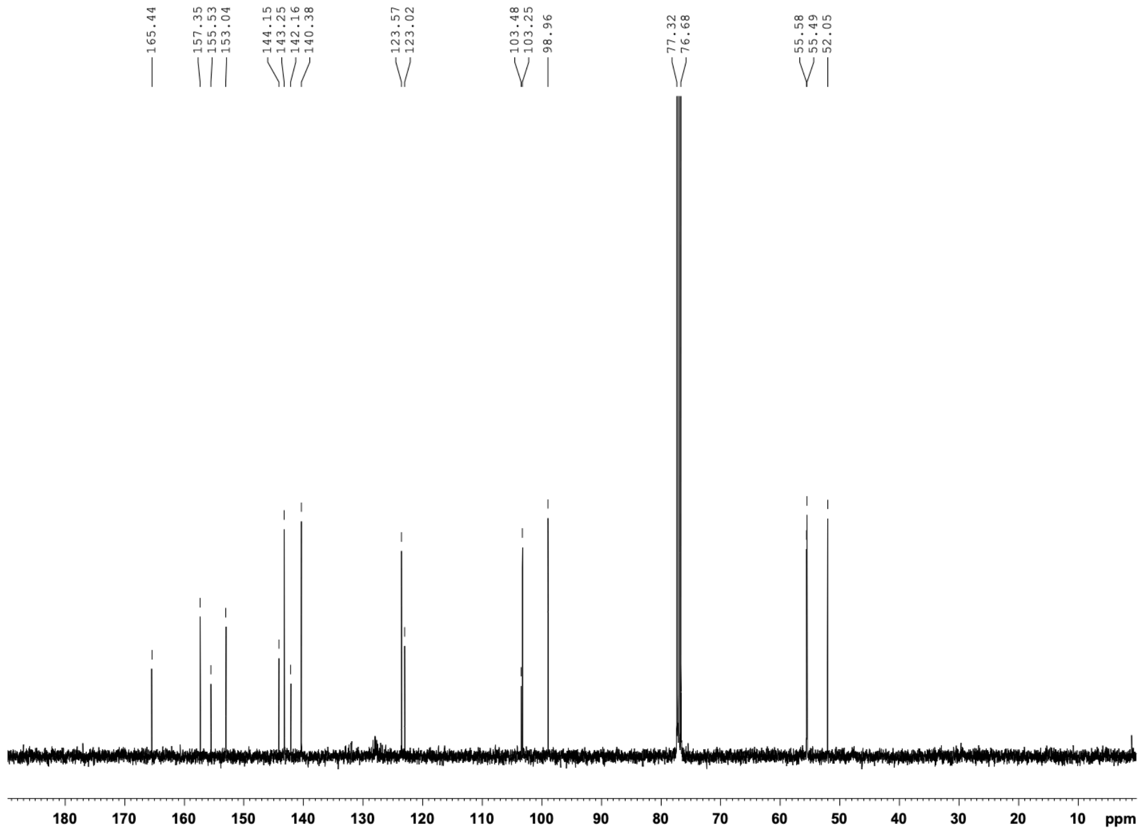

Methyl 7-[(3,4-dimethoxyphenyl)amino]thieno[2,3-*b*]pyrazine-6-carboxylate 2f

^{1}H NMR ($CDCl_3$, 400 MHz) of compound **2f**

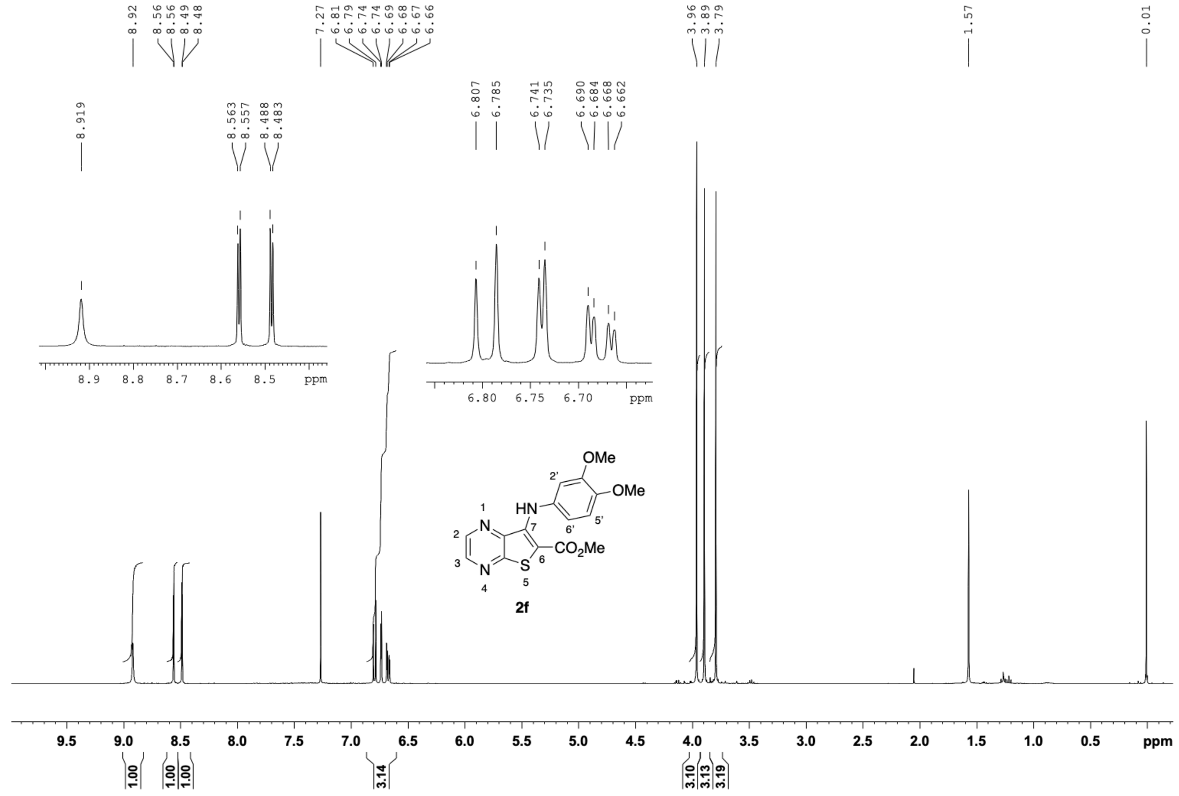

^{13}C NMR ($CDCl_3$, 100.6 MHz) of compound **2f**

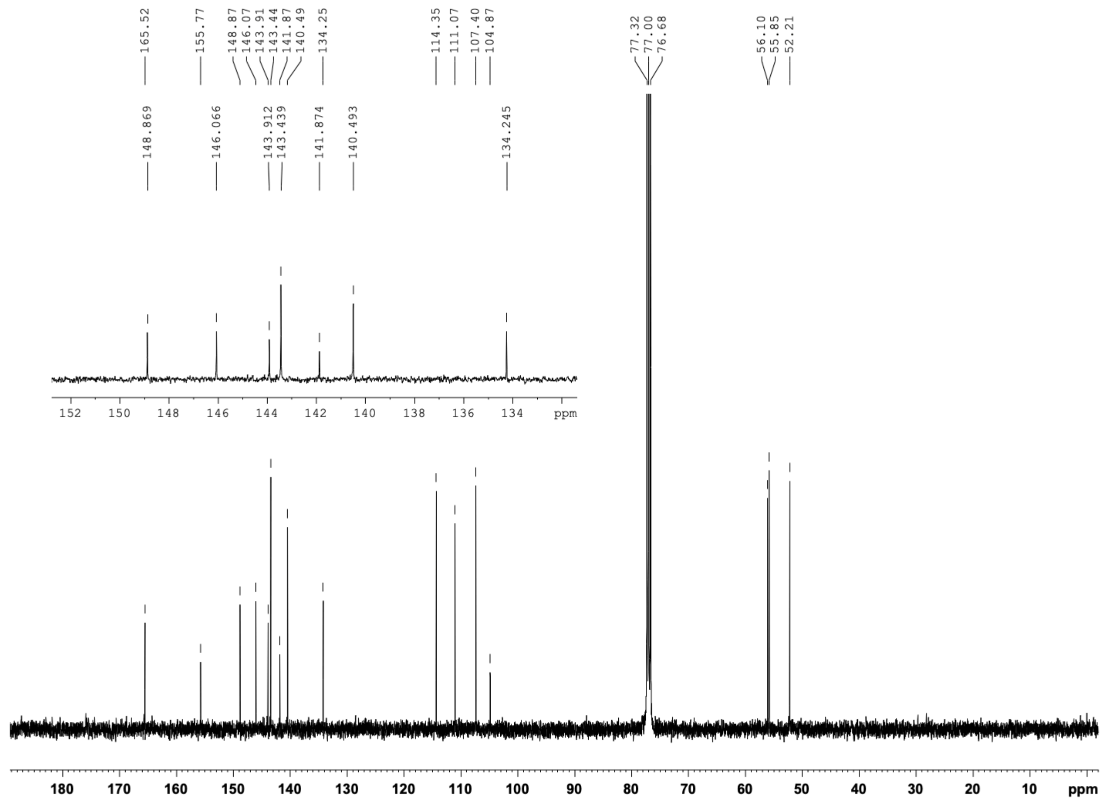

Methyl 7-[(3,5-dimethoxyphenyl)amino]thieno[2,3-*b*]pyrazine-6-carboxylate 2g

^{1}H NMR ($CDCl_3$, 400 MHz) of compound **2g**

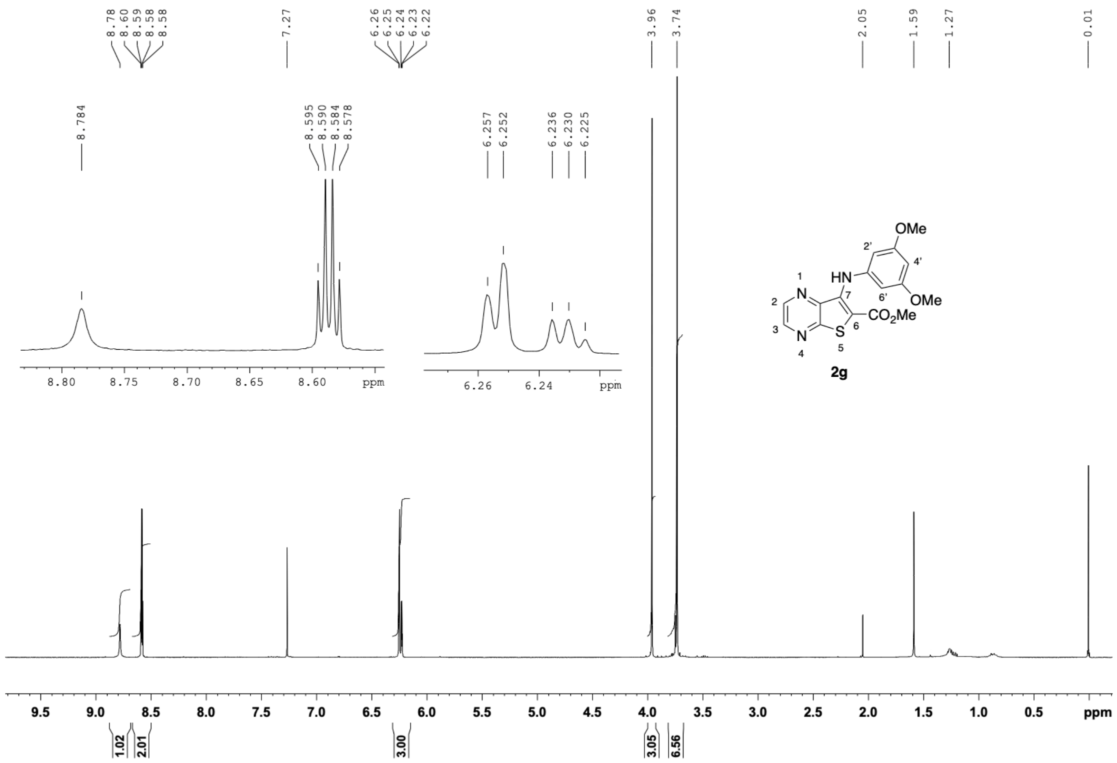

^{13}C NMR ($CDCl_3$, 100.6 MHz) of compound **2g**

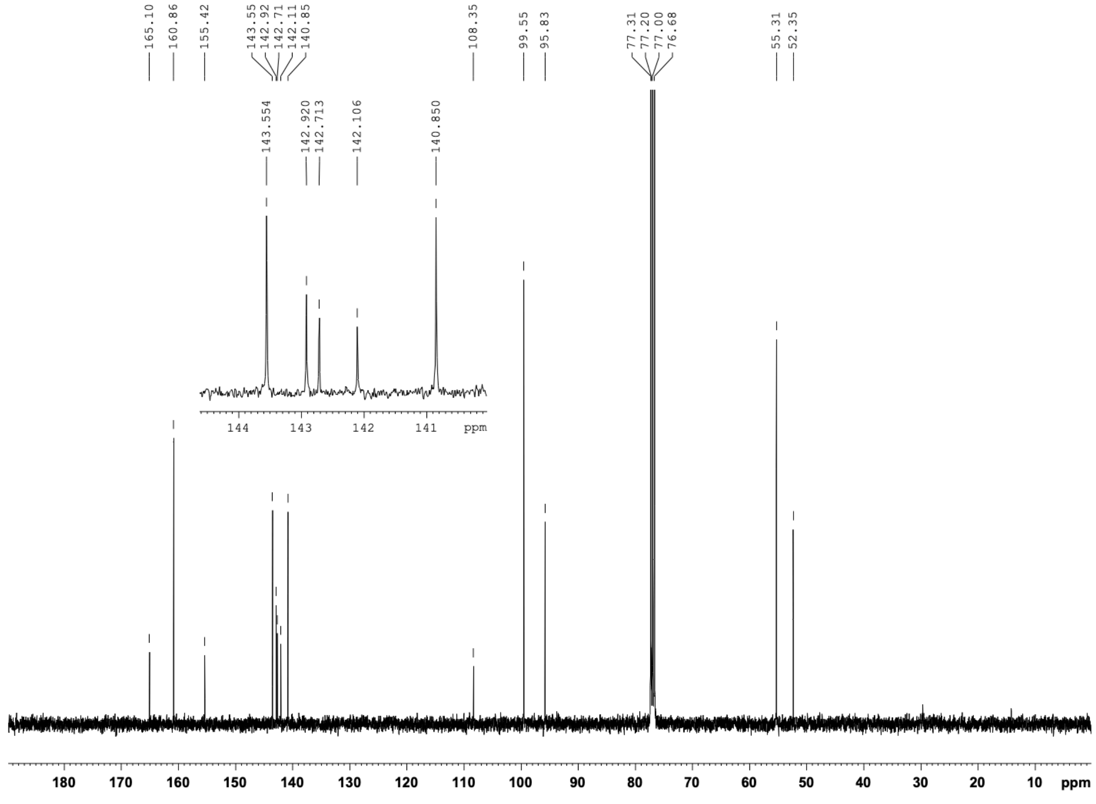

Methyl 7-[(3,4,5-trimethoxyphenyl)amino]thieno[2,3-*b*]pyrazine-6-carboxylate 2h

^{1}H NMR (DMSO-d_6, 400 MHz) of compound **2h**

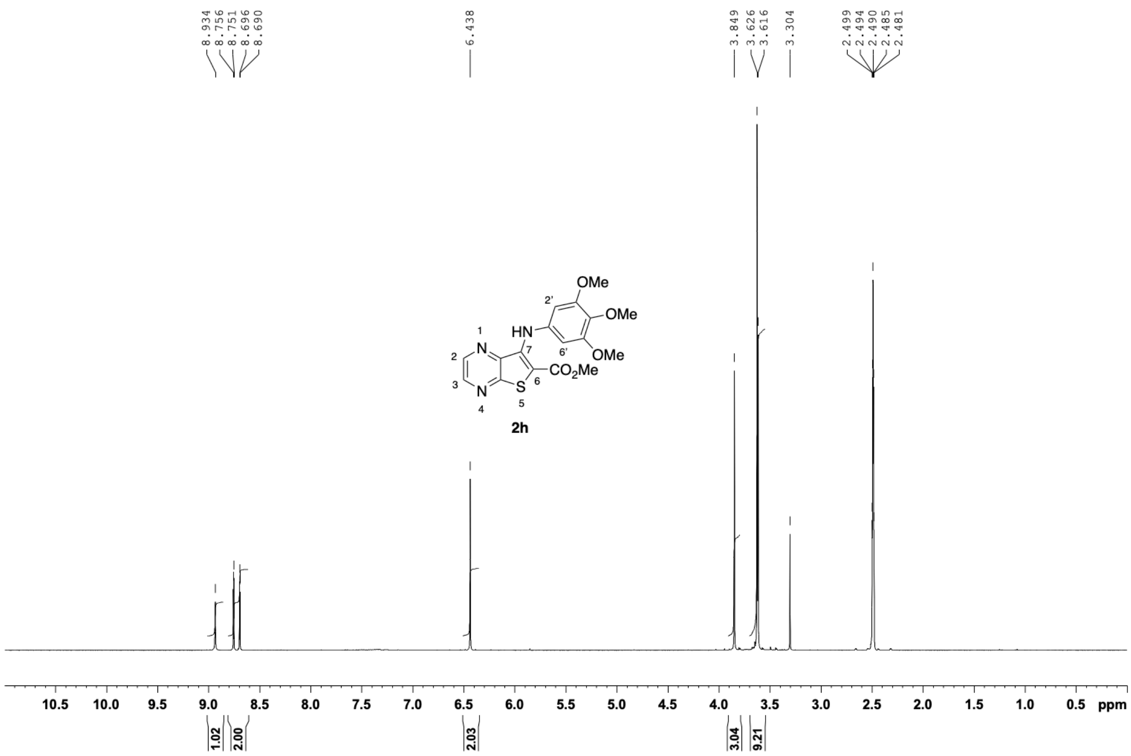

^{13}C NMR (DMSO-d_6, 100.6 MHz) of compound **2h**

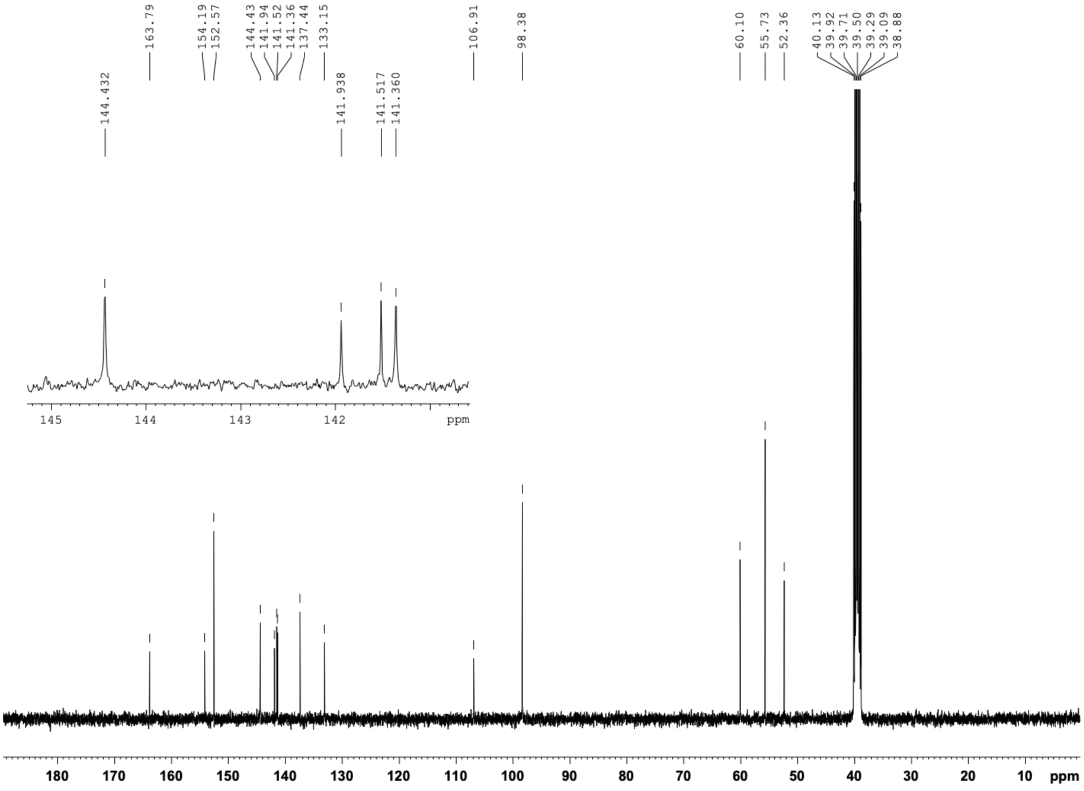

Methyl 7-[(2-fluorophenyl)amino]thieno[2,3-*b*]pyrazine-6-carboxylate 2i

^{1}H NMR ($CDCl_3$, 400 MHz) of compound **2i**

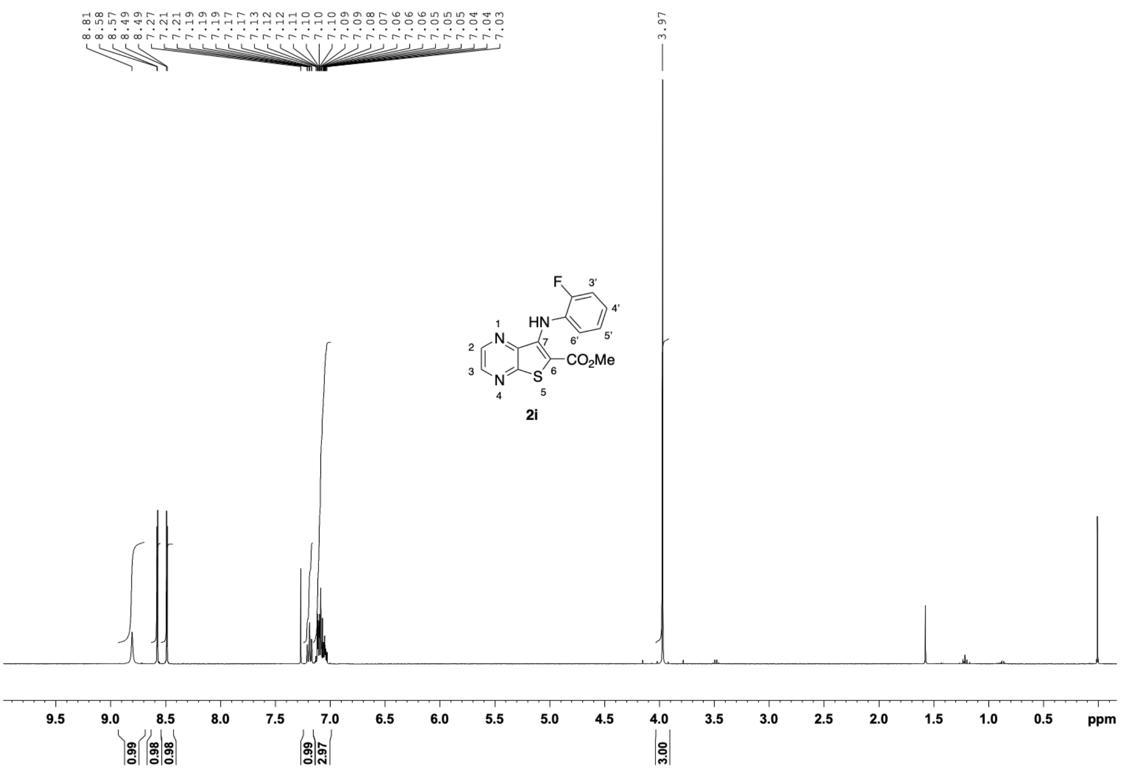

^{13}C NMR ($CDCl_3$, 100.6 MHz) of compound **2i**

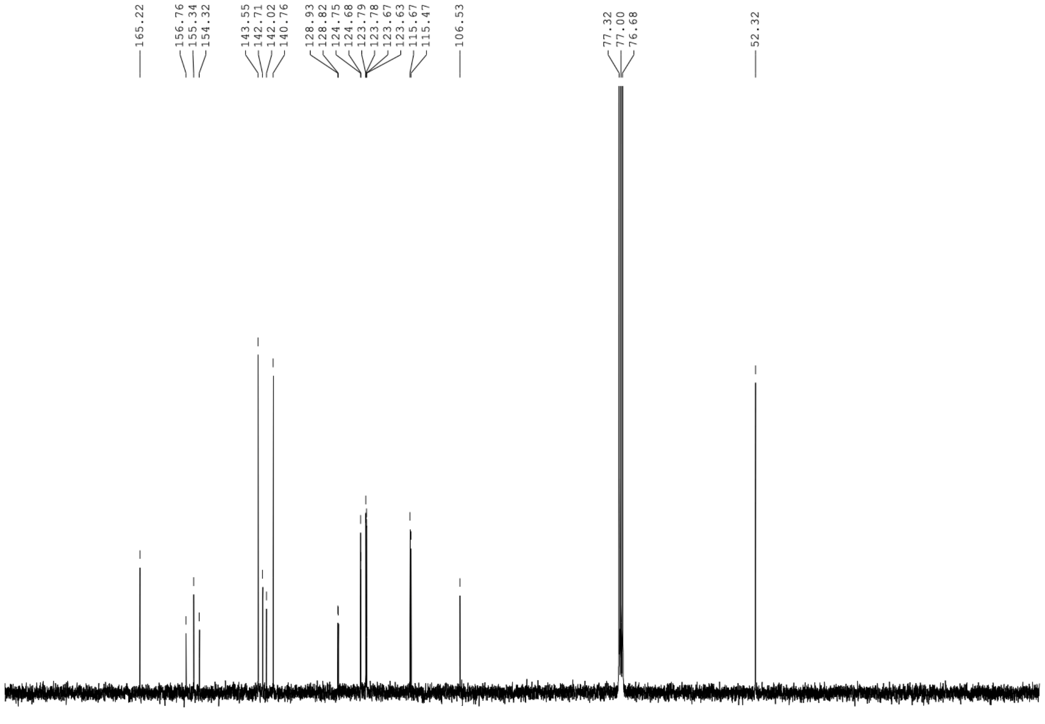

^{19}F NMR ($CDCl_3$, 376.48 MHz) of compound **2i**

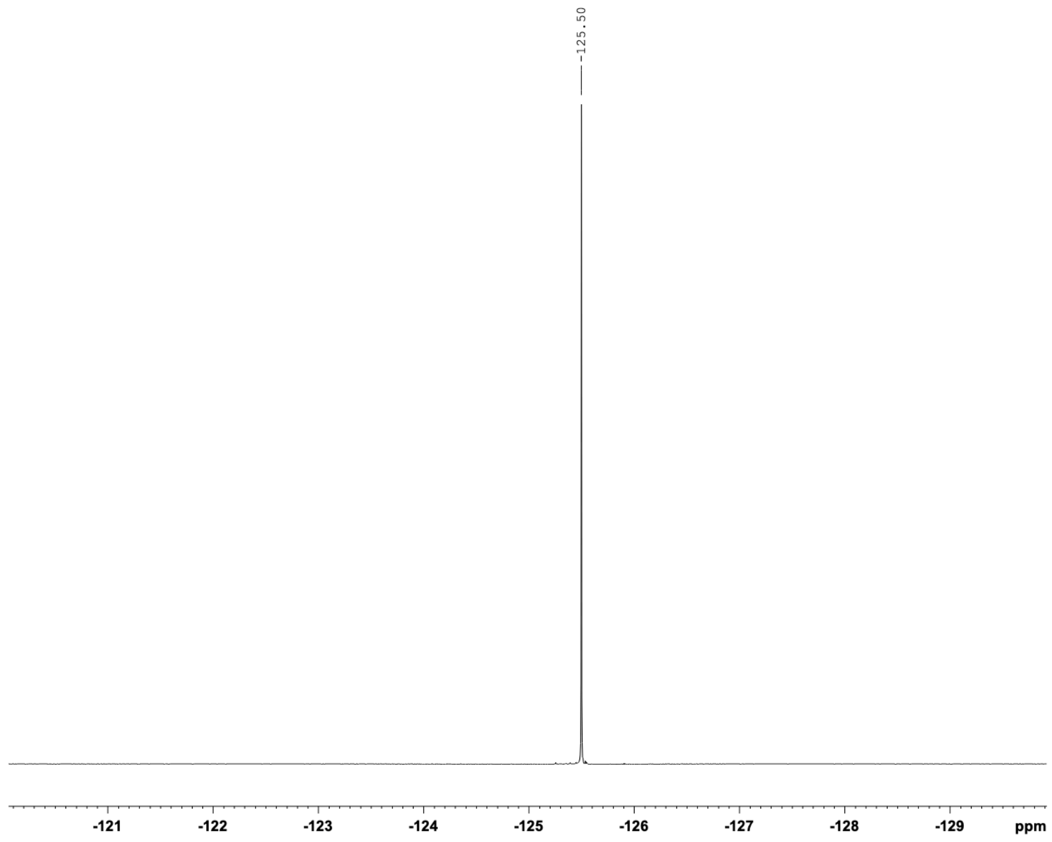

Methyl 7-[(3-fluorophenyl)amino]thieno[2,3-*b*]pyrazine-6-carboxylate 2j

^{1}H NMR ($CDCl_3$, 400 MHz) of compound **2j**

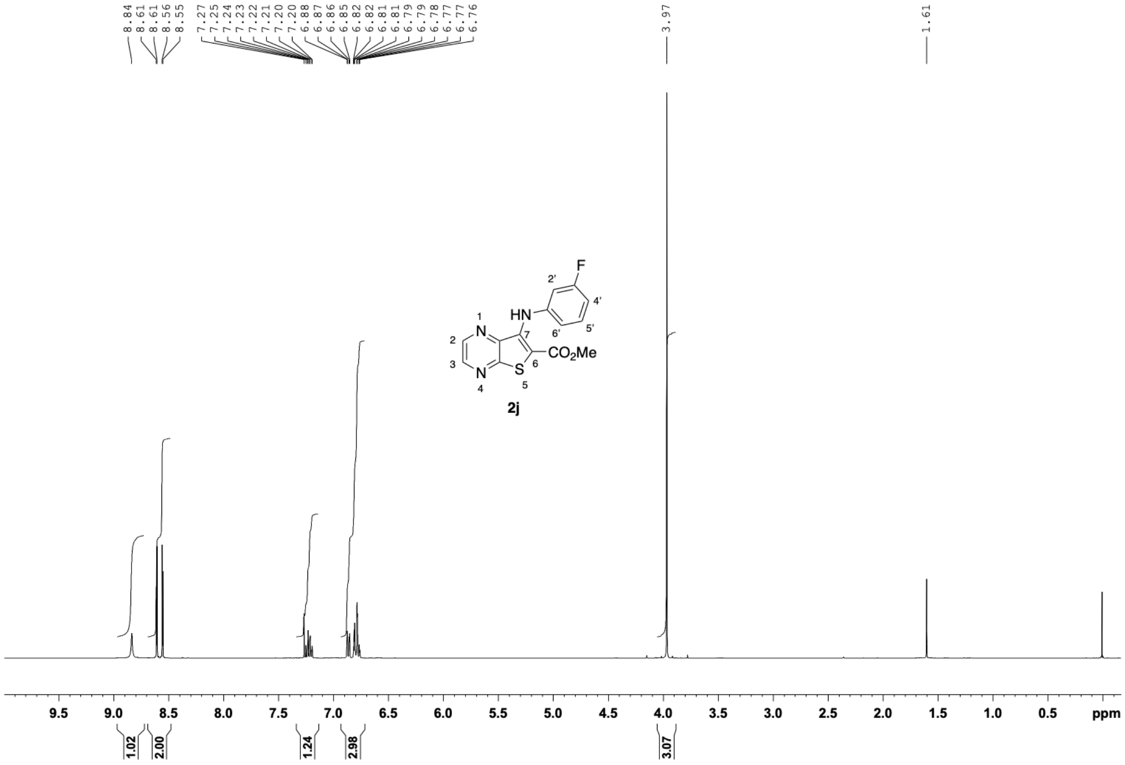

^{13}C NMR ($CDCl_3$, 100.6 MHz) of compound **2j**

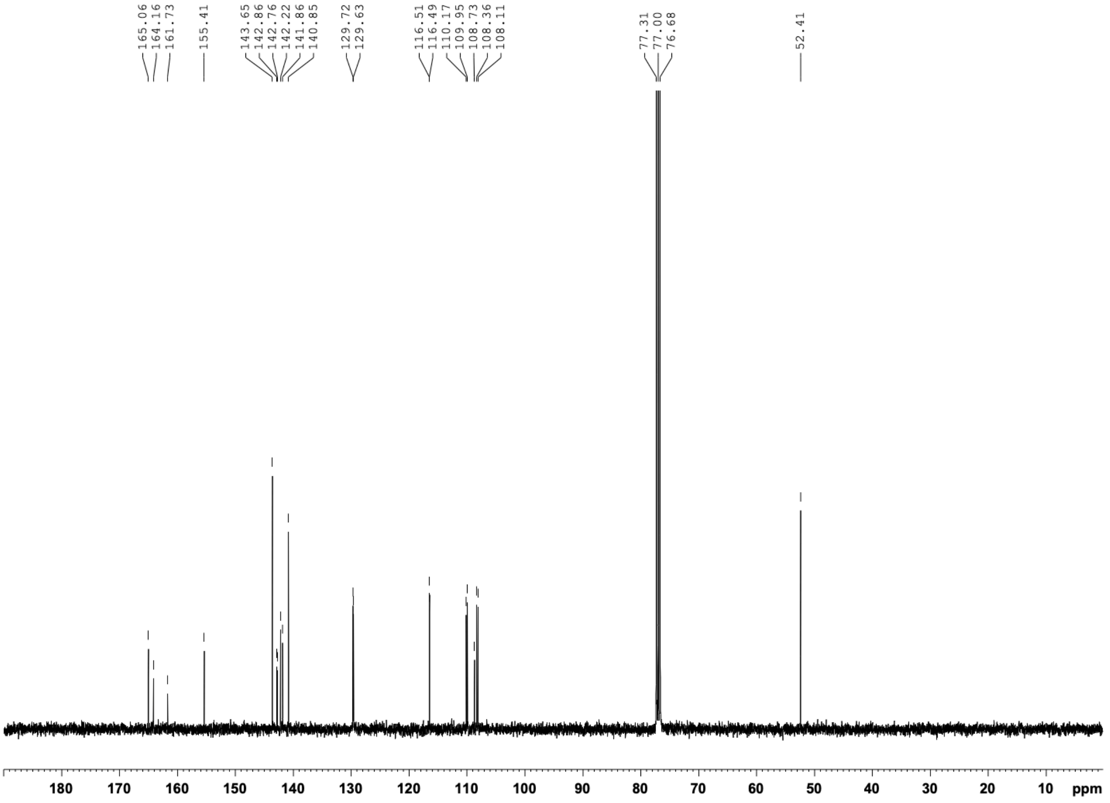

^{19}F NMR ($CDCl_3$, 376.48 MHz) of compound **2j**

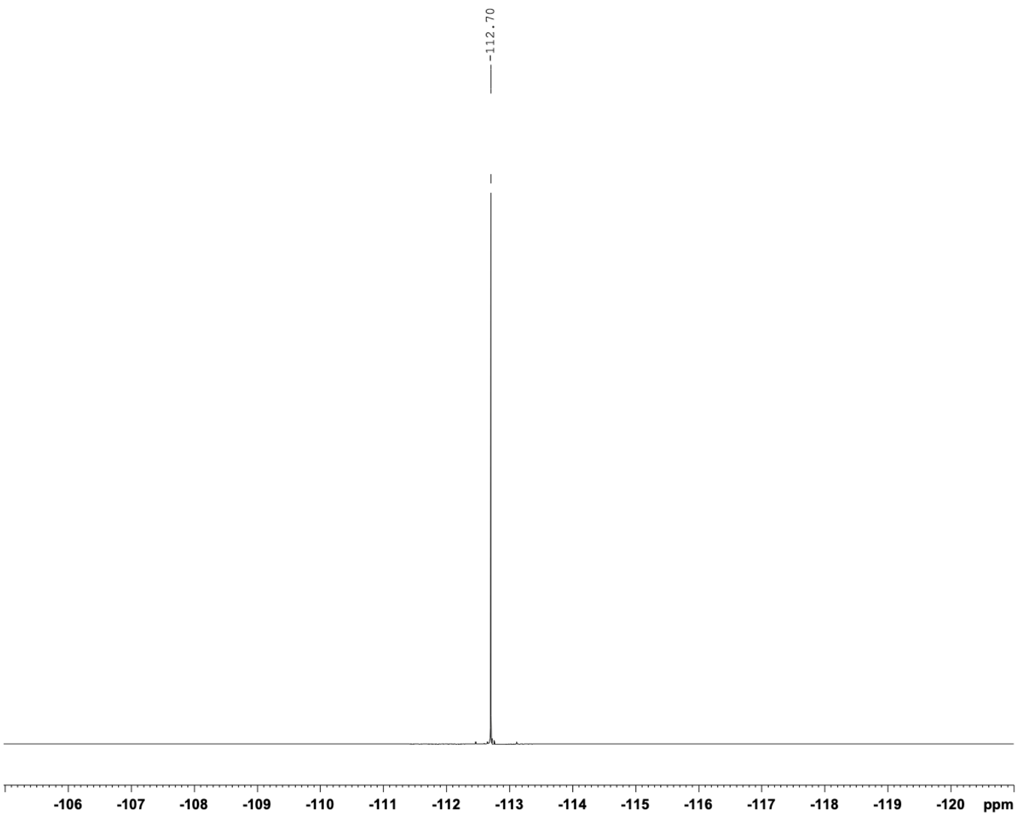

Methyl 7-[(4-fluorophenyl)amino]thieno[2,3-*b*]pyrazine-6-carboxylate 2k

^{1}H NMR (DMSO-*d*6, 400 MHz) of compound **2k**

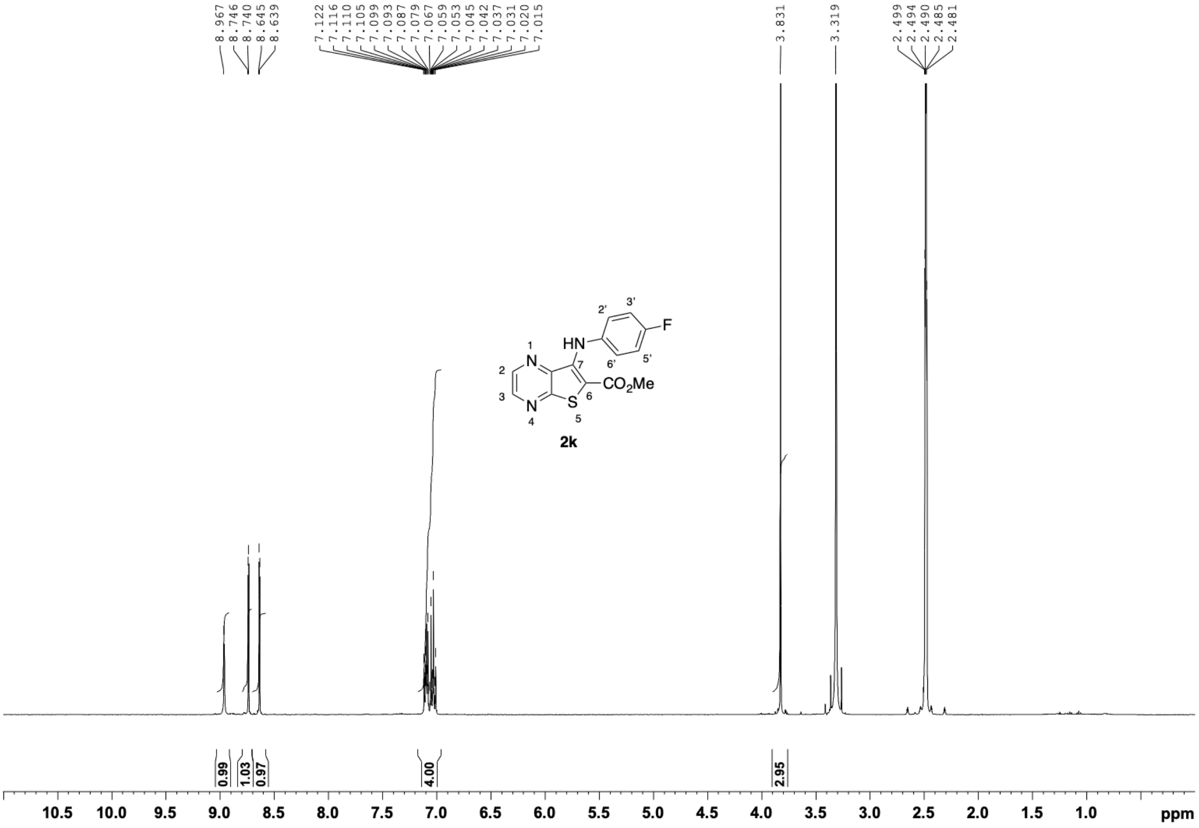

^{13}C NMR (DMSO-*d*6, 100.6 MHz) of compound **2k**

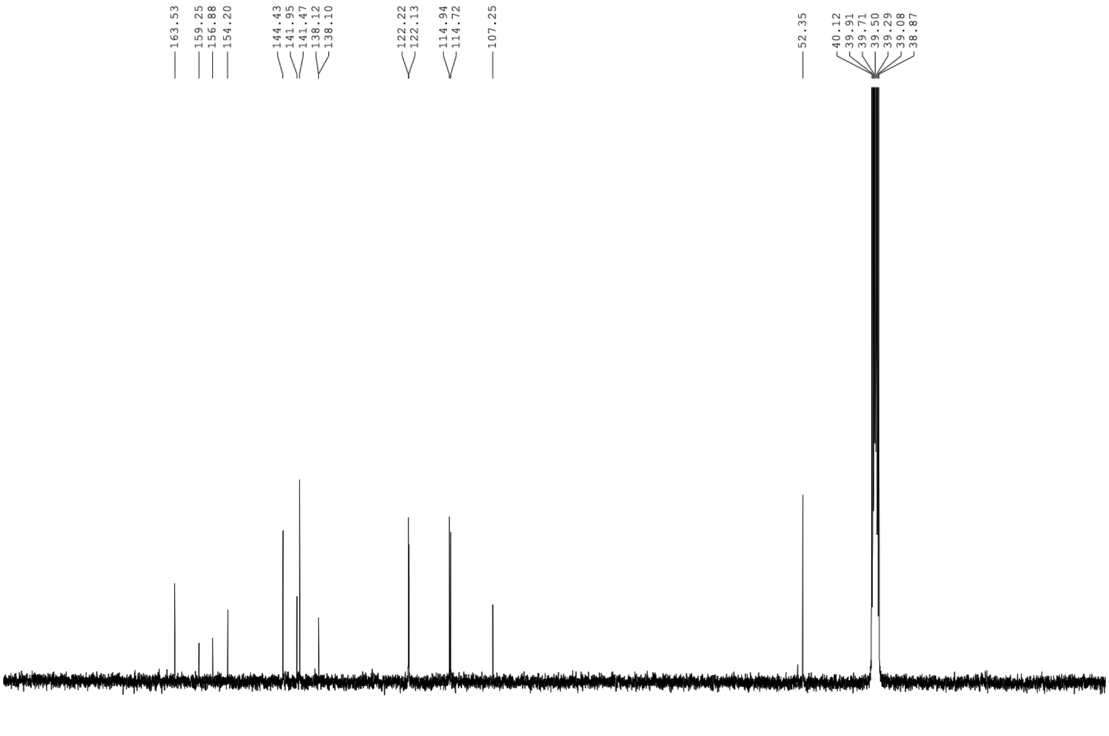

^{19}F NMR (DMSO-d_6, 376.48 MHz) of compound **2k**

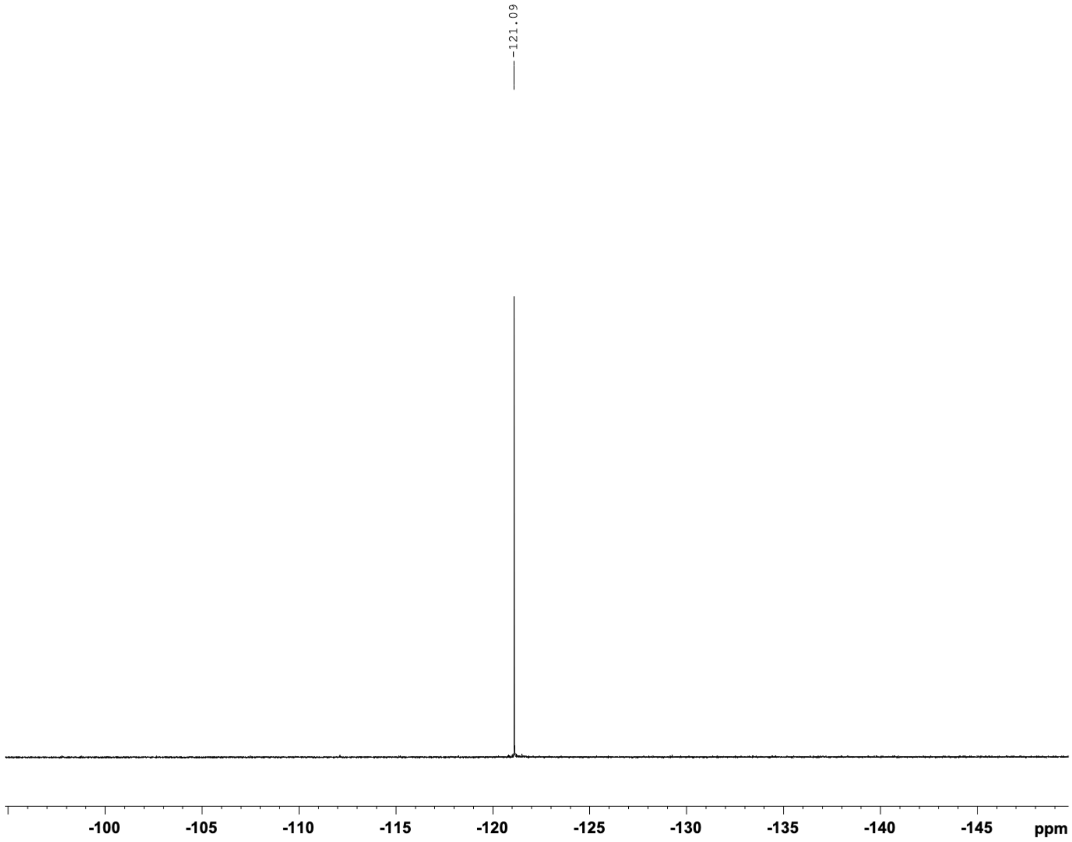

Methyl 7-(pyridin-3-ylamino)thieno[2,3-*b*]pyrazine-6-carboxylate 2l

^{1}H NMR ($CDCl_3$, 400 MHz) of compound **2l**

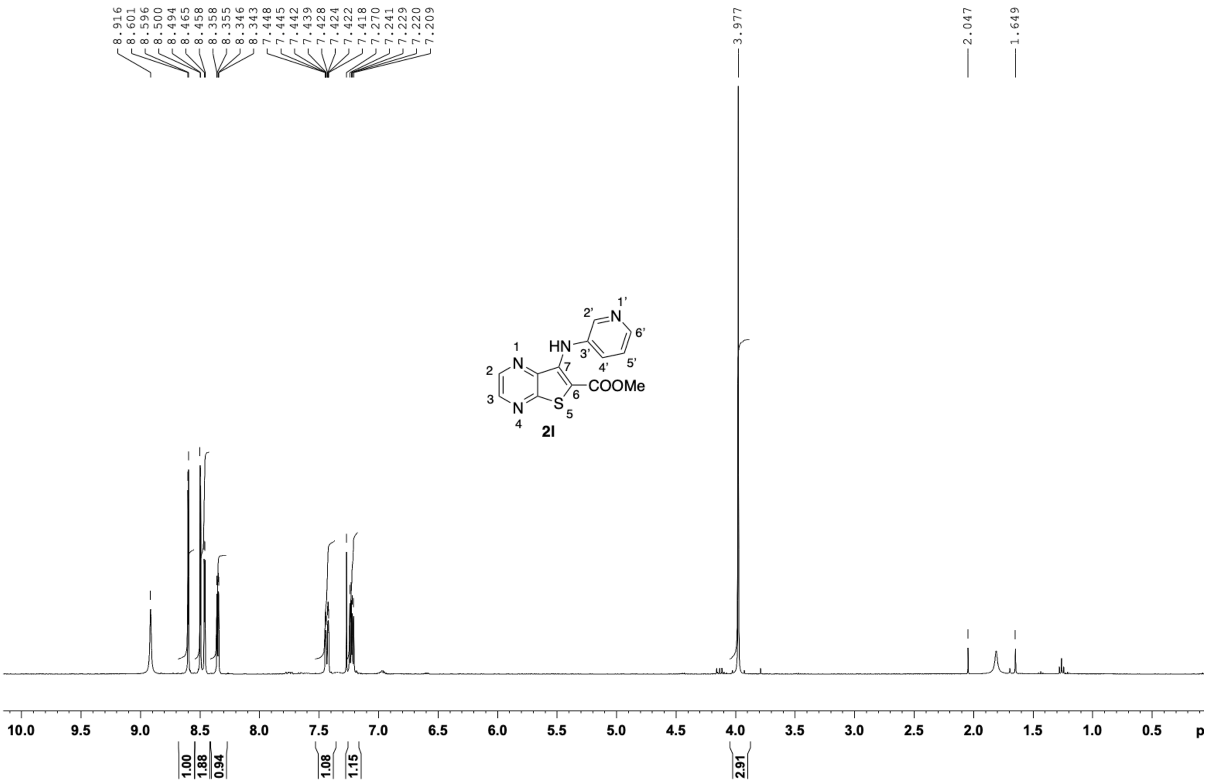

^{13}C NMR ($CDCl_3$, 100.6 MHz) of compound **2l**

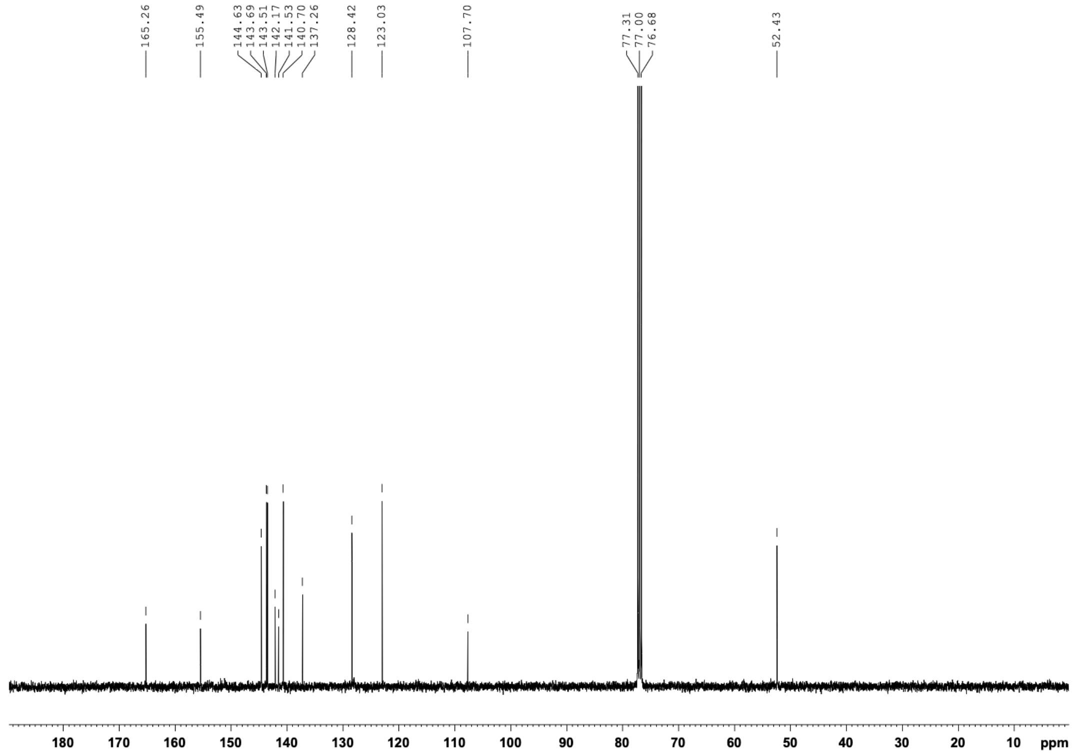

Methyl 7-(1*H*-pyrrol-1-yl)thieno[2,3-*b*]pyrazine-6-carboxylate 2m

^{1}H NMR ($CDCl_3$, 400 MHz) of compound **2m**

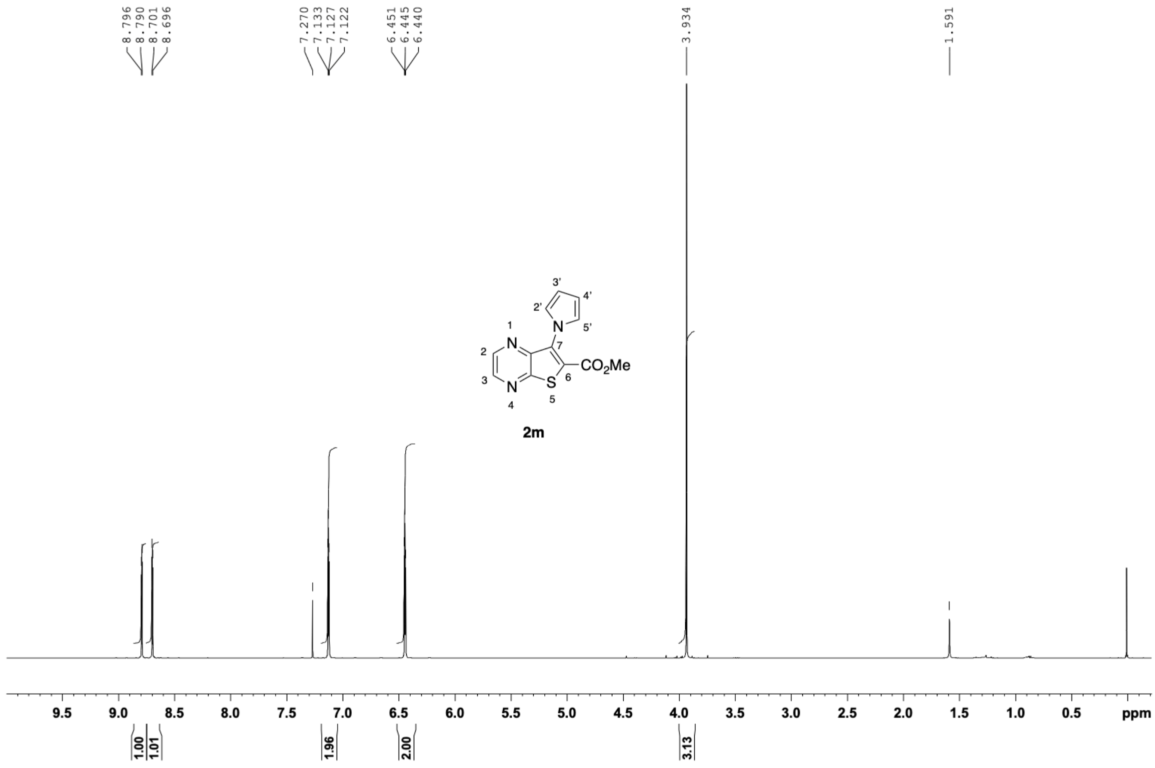

^{13}C NMR ($CDCl_3$, 100.6 MHz) of compound **2m**

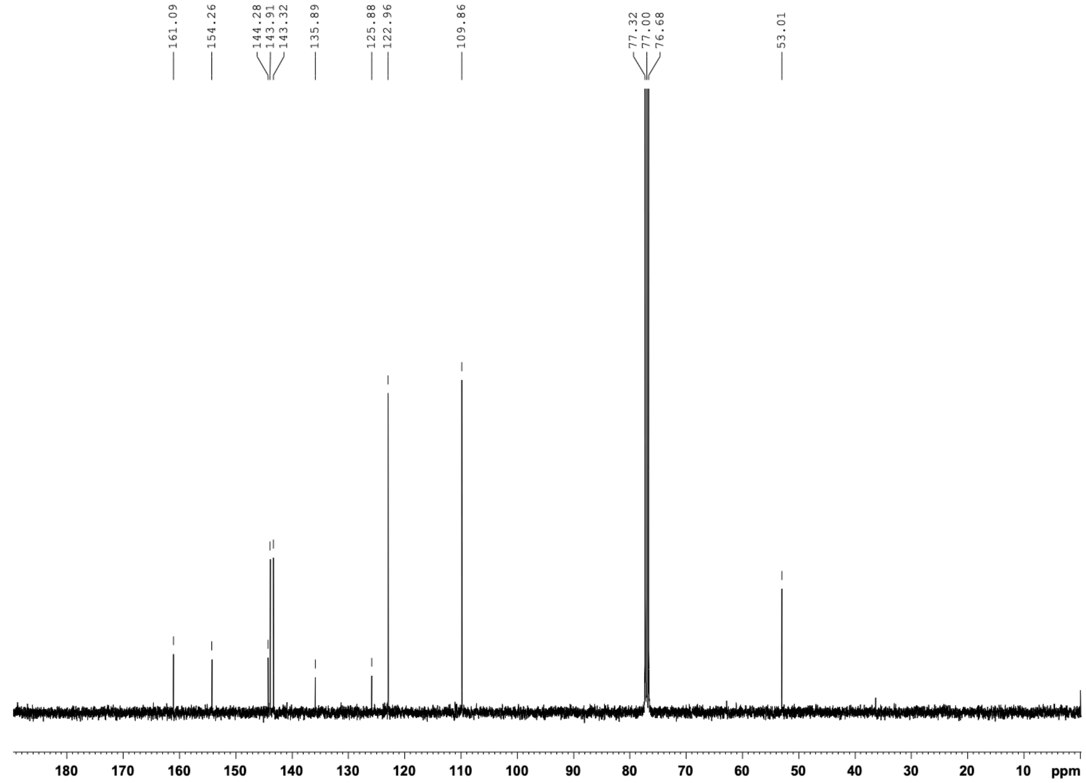

Methyl 7-[(4-cyanophenyl)amino]thieno[2,3-*b*]pyrazine-6-carboxylate 2n

^{1}H NMR ($CDCl_3$, 400 MHz) of compound **2n**

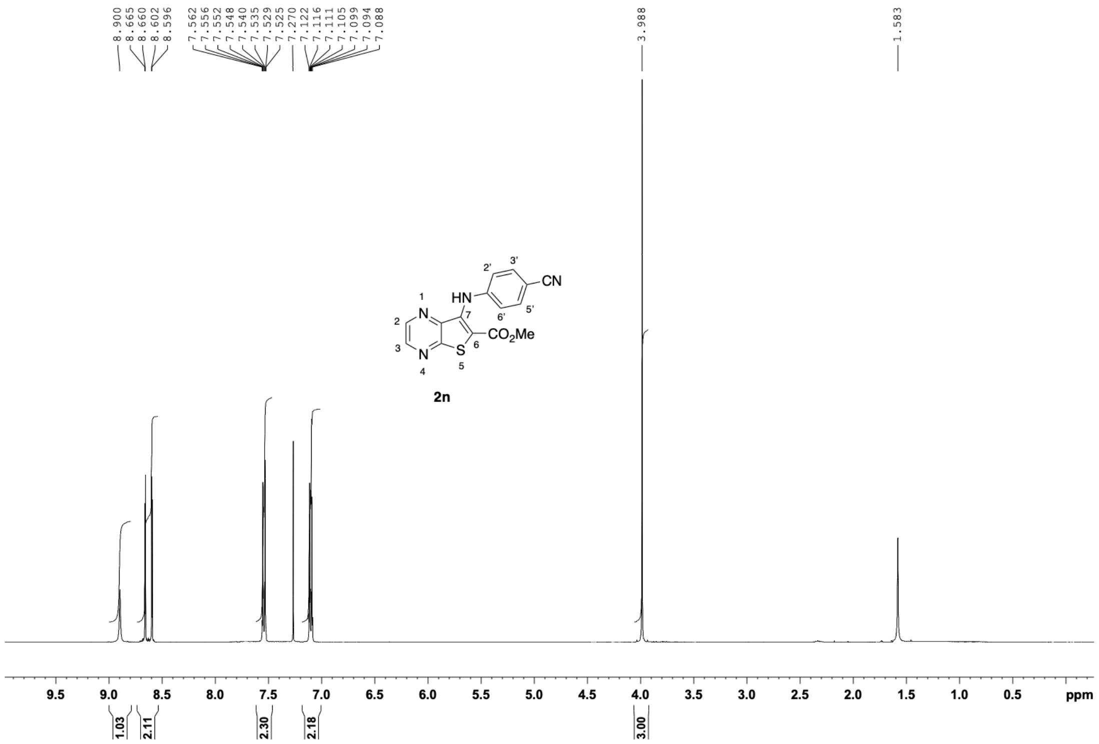

^{13}C NMR ($CDCl_3$, 100.6 MHz) of compound **2n**

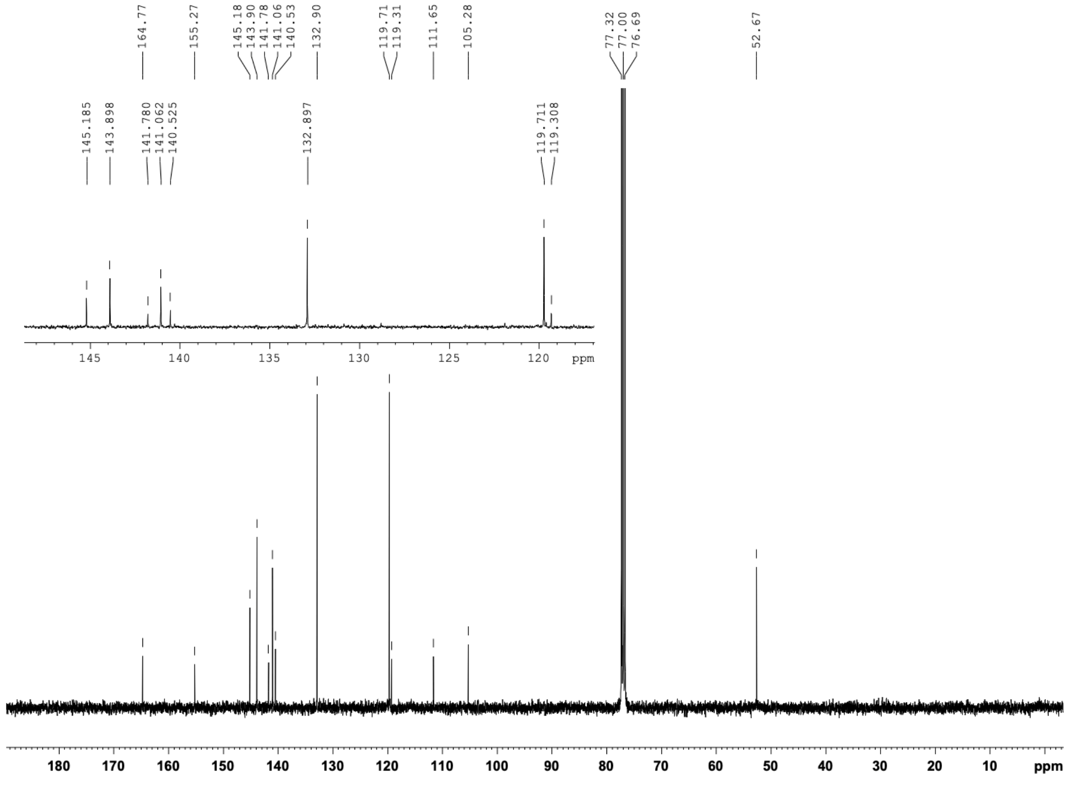

Methyl 7-[(2-nitrophenyl)amino]thieno[2,3-*b*]pyrazine-6-carboxylate 2o

^{1}H NMR ($CDCl_3$, 400 MHz) of compound **2o**

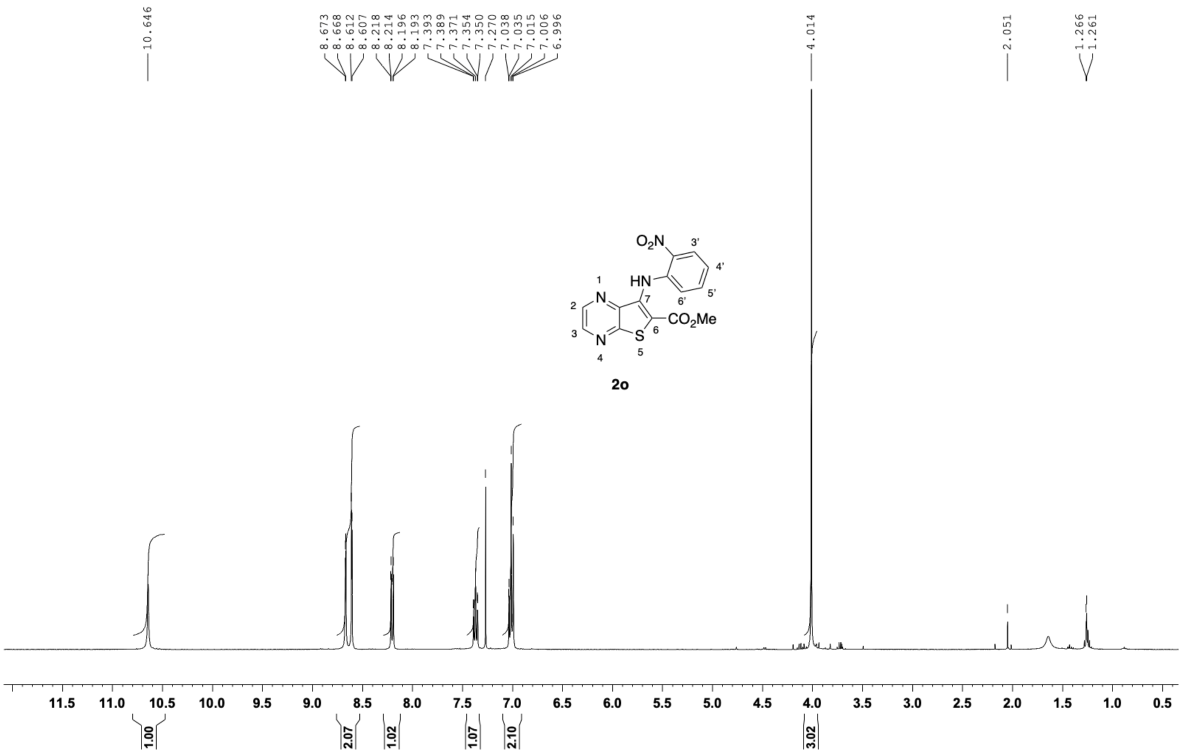

^{13}C NMR ($CDCl_3$, 100.6 MHz) of compound **2o**

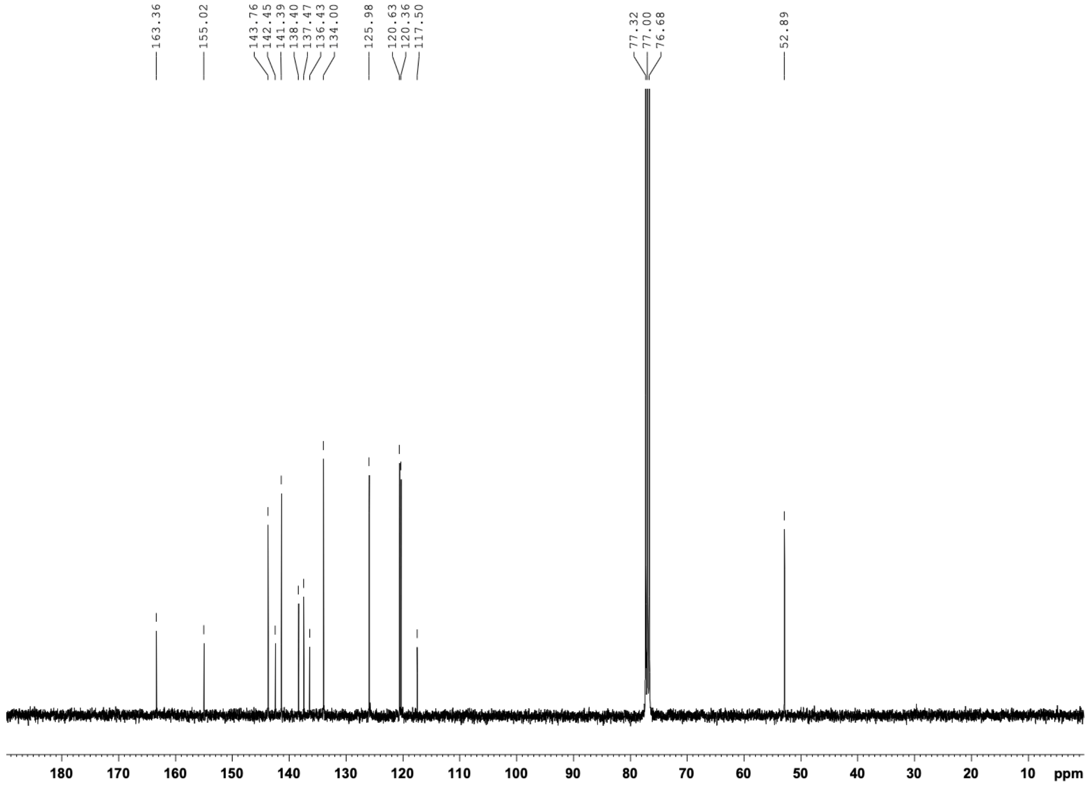

Supplement: Supplementary file 1 [file molecules-26-04823-s001.zip › molecules-1307054-supplementary.pdf]
